# Rapid regional perturbations to the recent global geomagnetic decay revealed by a new Hawaiian record

**DOI:** 10.1038/ncomms3727

**Published:** 2013-10-31

**Authors:** L. V. de Groot, A. J. Biggin, M. J. Dekkers, C. G. Langereis, E. Herrero-Bervera

**Affiliations:** 1 Paleomagnetic laboratory Fort Hoofddijk, department of Earth Sciences, Utrecht University, Budapestlaan 17, 3584 CD Utrecht, The Netherlands; 2 Geomagnetism Laboratory, Oliver Lodge Labs, School of Environmental Sciences, University of Liverpool, Oxford Street, Liverpool L69 7ZE, UK; 3 Hawaii Institute of Geophysics and Planetology, University of Hawaii at Manoa, 1680 East-West Road, POST 602, Honolulu, Hawaii 96822, USA

## Abstract

The dominant dipolar component of the Earth’s magnetic field has been steadily weakening for at least the last 170 years. Prior to these direct measurements, archaeomagnetic records show short periods of significantly elevated geomagnetic intensity. These striking phenomena are not captured by current field models and their relationship to the recent dipole decay is highly unclear. Here we apply a novel multi-method archaeomagnetic approach to produce a new high-quality record of geomagnetic intensity variations for Hawaii, a crucial locality in the central Pacific. It reveals a short period of high intensity occurring ~1,000 years ago, qualitatively similar to behaviour observed 200 years earlier in Europe and 500 years later in Mesoamerica. We combine these records with one from Japan to produce a coherent picture that includes the dipole decaying steadily over the last millennium. Strong, regional, short-term intensity perturbations are superimposed on this global trend; their asynchronicity necessitates a highly non-dipolar nature.

## Abstract (web summary)

The Earth’s geomagnetic field has weakened in recent centuries, leading to calls for historic reconstructions; however, records are sparse and unevenly distributed. de Groot *et al.* provide a new, high-quality record from Hawaiian lavas, revealing crucial insights into past geomagnetic field fluctuations.

## Graphical abstract

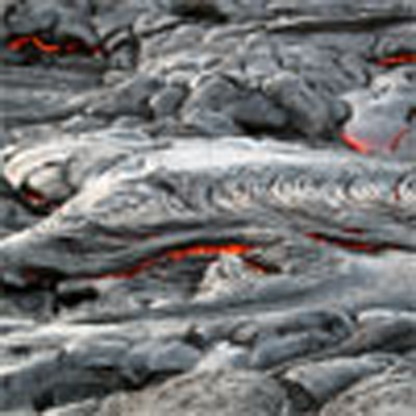

Evidence that the geomagnetic field has exhibited numerous intense, short-lived, regional maxima in intensity at various times and locations in the last few thousand years is rapidly accumulating: records from Europe^1^,^2^, Mesoamerica^3^,^4^, West Africa^5^ and the Middle-East^6^,^7^,^8^ show at least one intensity high. These highly intriguing features are poorly understood because the limited number and uneven geographical distribution of reliable archaeointensity records hamper a meaningful analysis. Current methods to obtain absolute archaeointensities and palaeointensities are only applicable to materials that acquired a thermal remanent magnetization by cooling in the Earth’s magnetic field. Until now, only records using well-dated man-made baked artefacts such as pottery, kilns and copper slags attained the temporal resolution needed to reveal the occurrence of short-lived rapid fluctuations in geomagnetic field intensity. Archaeological artefacts, however, are only available for specific ages and locations linked to ancient civilizations—the majority of data being derived from Europe, the Middle East and Mesoamerica. To provide spatial and temporal constraints on the occurrence of archaeomagnetic intensity highs and to assess the driving mechanism causing these short-lived phenomena, we have to turn to more widely available extrusive rocks. However, as a consequence of their differing composition and thermal history, the success rate in obtaining reliable estimates of the geomagnetic field intensity is much lower for lavas than for archaeological artefacts.

The island of Hawaii (USA) is a particularly important region to obtain a regional curve of geomagnetic field behaviour from because of its location in the central Pacific. Without reliable full vector data from Hawaii, a vast region is not adequately covered in geomagnetic models. Fortunately, numerous well-dated lava flows were emplaced at a relatively high frequency, making it possible to produce curves with high temporal resolution. The palaeointensity of Hawaii is well studied^9^,^10^,^11^,^12^; however, variations between near-coeval flows are unacceptably large (30–50 μT), testifying to the difficulties associated with obtaining meaningful estimates from lavas.

Here we report the results of archaeointensity experiments performed on samples from well-dated Hawaiian flows using a combination of methodologies. Our multi-method archaeointensity approach and the very strict selection criteria applied to both the data obtained in this study and existing data produce a coherent record of archaeointensity variations for Hawaii for the past ~1,800 years. This record is demonstrated to be robust through the consistency of results produced using very different methods and the agreement with the observational record over the last 170 years. The unusually high temporal resolution in our record is primarily attained by calibrating a non-heating palaeointensity approach—the pseudo-Thellier technique^13^. We show that this method reconstructs the intensity of the palaeofield reliably from most of the samples that are unsuccessful in conventional archaeointensity methods because of thermally induced alteration. The new Hawaiian archaeointensity record indicates notably high values from ~1000–1150 AD followed by an undulating decrease thereafter. Similar phenomena are observed in Europe 200 years earlier and in Mesoamerica 500 years later. A comparison with these and other records of geomagnetic field variations for the northern hemisphere implies a non-dipole character of such comparatively short-lived intensity highs. Furthermore, they cannot be explained by westward drift of a single geomagnetic phenomenon. We therefore conclude that these rapid regional variations in the intensity of the Earth’s magnetic field result from spatially and temporally non-uniform geomagnetic phenomena that are presently enigmatic in terms of their sources.

## Results

### Sampling

We sampled 33 independent cooling units on the Big Island of Hawaii at 43 different locations. At some locations, we sampled multiple sites, often at different depths in the flow. Details of all 63 sampled sites are provided in Supplementary Tables S1, S2. Reliable global estimates of the geomagnetic field intensity are available since 1840 AD^14^,^15^; the 37 sites taken from lavas younger than 1840 AD are therefore suitable to verify the archaeointensity methodologies and selection criteria used. The other 26 sites date between 430 and 1840 AD. Localities from before 1790 are radiocarbon-dated, with all dates used in this study originating from two studies^16^,^17^; we recalibrated these dates using the latest INTCAL.09-curve^18^,^19^.

We applied both thermal^20^,^21^ and microwave Thellier-type techniques^22^ and domain-state-corrected multispecimen archaeointensity experiments^23^,^24^ (MSP-DSC). Detailed information on all absolute archaeointensity experiments is provided in the Methods section. Furthermore, we calibrate a non-heating palaeointensity technique, the pseudo-Thellier approach^13^, to make it suitable to obtain absolute estimates of the intensity of the palaeofield from samples that are unsuccessful in conventional archaeointensity experiments.

### IZZI-Thellier experiments

In total, 96 samples distributed over all sampled cooling units were subjected to IZZI-Thellier archaeointensity determinations^21^,^25^,^26^. The results were grouped by their age and, if four or more samples passed our selection criteria (see Methods and Supplementary Table S3), their results were averaged. A further criterion was applied requiring that the s.d. of the accepted results was <20% of their mean. Out of 33 cooling units/age groups, just two reliable archaeointensity estimates were obtained and both had post-1840 AD ages (Supplementary Fig. S1, Supplementary Table S4).

### Microwave Thellier experiments

The microwave archaeointensity approach emulates the conventional IZZI-Thellier method but uses high frequency microwaves instead of thermal energy to (de)magnetize the samples^22^,^27^,^28^. Microwave archaeointensity experiments were performed using a combination of Thellier-type protocols on 23 samples from four key pre-1840 sites, with just one of these lavas yielding a reliable archaeointensity estimate (see Methods).

### Domain-state-corrected multispecimen experiments

Samples from eight of our 63 sites were subjected to MSP-DSC experiments^23^,^24^ by virtue of them passing the ‘ARM test’^29^ for thermally induced alteration. Owing to varying alteration temperatures, MSP-DSC experiments were performed at several different temperatures between 150 and 360 °C. Four of these eight sites were younger than 1840 AD and all these yielded the expected archaeointensity to within 3 μT—that is, accurate to better than 10%. (see Methods, Supplementary Fig. S2, Supplementary Table S5).

### Pseudo-Thellier technique

**Figure 1:**
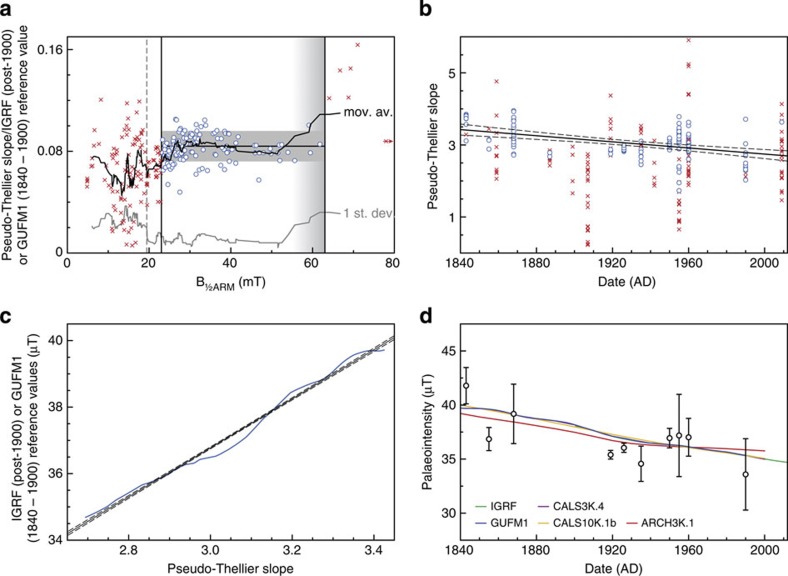
Pseudo-Thellier calibration. As the pseudo-Thellier slope linearly depends on the geomagnetic field a sample cooled in, the ratio of the pseudo-Thellier slope and its reference geomagnetic field intensity should yield a constant value for samples that behave equally. This ratio is on the vertical axis in **a**, the grain-size indicator—the AF strength that imparts half of the saturated ARM (B_½ARM_)—is on the horizontal axis. For samples with a B_½ARM_ between 23 and 63 mT (blue circles), the 10-point moving average (black line) is relatively constant, and s.d. of the 10-point moving average (grey line) is relatively low. Furthermore, the s.d. of the mean of all data within this B_½ARM_ domain is <15% of that mean; therefore, these samples are accepted for further analyses. Data outside this domain are shown as red crosses (one samples’ B_½ARM_ is >80 mT, depicted by a red arrow). All pseudo-Thellier factors of the samples included in **a** are plotted as function of their age in **b**, with the same symbols (rejected data red crosses, accepted data blue circles). The linear fit through the accepted data points (the solid line, dashed lines: one-s.d.) shows a decaying trend, fully in line with the relatively constant decay of the geomagnetic field intensity from direct observations. This linear fit is then calibrated on the observed decaying trend in GUFM1 (pre-1900) and IGRF (post-1900) reference values (blue line, **c**) yielding a linear calibration relation (black solid line) with one-s.d. error envelope (dashed lines) to convert pseudo-Thellier values to absolute archaeointensity estimates. After averaging all accepted data per age group, the pseudo-Thellier results (open black circles) with their one-s.d. interval (vertical error bars) yield the trends observed in the different geomagnetic models (**d**); and are all within 3 μT of their expected values.

**Figure 2:**
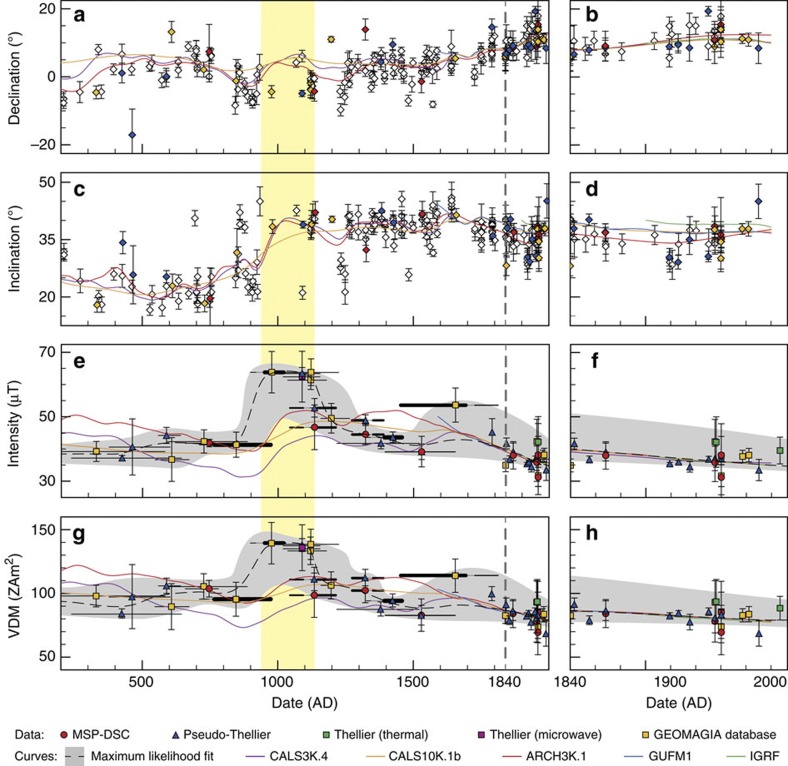
Full vector geomagnetic variations at Hawaii for the past ~1,800 years. The right-hand panels are rescaled from the left-hand panels to detail the period between 1840 and 2010 AD (right of the grey dashed line in the left-hand panels). **a**,**b** and **c**,**d** give the declination and inclination, respectively. Open diamonds are data from the GEOMAGIA database for which only directional information is available. The coloured declinations and inclinations in these panels originate from the same sites as the intensity estimates in **e**,**f** and **g**,**h** with the same colour and age. The vertical error bars in **a**–**d** are the α95 confidence intervals associated with the palaeodirection. Archaeointensities obtained by various methods are depicted with different symbols in **e**–**h**: MSP-DSC in red circles; pseudo-Thellier in blue triangles; thermal IZZI-Thellier in green squares; microwave Thellier in purple squares; and previously reported archaeointensities from the GEOMAGIA database in yellow squares. The vertical error bars in the intensity data (**e**,**f**) are the one-s.d. error envelopes obtained from the archaeointensity methods. The errors on the VDM’s (**g**,**h**) are calculated by combining the shallowest possible α95 inclination with the lower bound of the one-s.d. of the archaeointensity and *vice versa*, per site. Interpolated maximum likelihood curves are shown as dashed black lines with their one-s.d. as a shaded grey area in the intensity and VDM in **e**–**h**. Reference models are shown as solid coloured lines: CALS3K.4 in purple; CALS10K.1b in yellow; ARCH3K.1 in red; GUFM1 in blue; and IGRF in green. The period of high archaeointensities between ~950 and ~1150 AD is tentatively indicated by the yellow area. All ^14^C datings were recalibrated using INTCAL.09. For clarity, error bars in age are omitted from the directions in **a**–**d**; in **e**,**g**, one-s.d. intervals are shown as horizontal bars. If more than one one-s.d. age range was obtained from the recalibration, they are shown as thicker black bars connected by a thin grey line. The thickness of the solid black bars is proportional to their relative likelihood.

Owing to our strict selection criteria, we obtained a reliable estimate of the palaeofield’s intensity for only nine of our 33 possible age groups using conventional archaeointensity methods. A complicating issue with these lavas is that the heating of the samples in conventional archaeointensity experiments frequently induces alteration, rendering the obtained result meaningless. Relative palaeointensity records are frequently obtained from sediments. In relative palaeointensity techniques, the natural remanent magnetization (NRM) of the samples is compared with (progressively increasing) imparted anhysteretic remanent magnetizations (ARMs), thus avoiding heating the samples. The relative pseudo-Thellier technique^13^ (see Methods) is analogous to the thermal Thellier technique: it compares the demagnetized NRM and the acquired ARM for increasingly higher alternating field (AF) values in an Arai plot (Supplementary Fig. S3). Yu *et al.*^30^ explored the potential of using the pseudo-Thellier method on lavas and found that its outcome is dependent on the grain-size distribution of the remanence-carrying grains. To select the pseudo-Thellier results that can be reliably compared, we use a selection criterion based on the grain-size indicator B_½ARM_, the magnitude of the AF for which half of the maximum ARM is imparted—a parameter that can be obtained directly from the pseudo-Thellier experiments. For the samples younger than 1840 AD, the obtained pseudo-Thellier slopes are divided by the age-equivalent reference values from the GUFM1 (pre-1900) and IGRF (post-1900) models; this ratio is then plotted against the samples’ B_½ARM_ (Fig. 1a). As the pseudo-Thellier slope is linear with the Earth’s magnetic field the samples cooled in, this ratio should yield a constant value for samples with similar pseudo-Thellier behaviour. To assess the feasibility of including samples as a function of B_½ARM_, the 10-point moving average and its s.d. are determined (Fig. 1a). The s.d. of the moving average of the ratio is a measure of scatter among the samples. It reveals a high degree of scatter for samples with B_½ARM_s <19.5 mT; and the moving average only becomes constant for samples with a B_½ARM_ >23 mT. We therefore choose 23 mT as the lower limit of the B_½ARM_ window of reliable samples. Fewer data are available to define the upper limit of this window; however, for samples with a B_½ARM_ between 23 and 63 mT, the s.d. of the mean of the ratio is <15% of that mean. Thus, we conclude that samples with a B_½ARM_ between 23 and 63 mT can be safely utilized. For all samples younger than 1840 AD with a B_½ARM_ between 23 and 63 mT, the pseudo-Thellier slopes show a decreasing trend with age (Fig. 1b). We fit the linear trend line through the accepted pseudo-Thellier slopes on the decreasing trend in the GUFM1 and IGRF models (Fig. 1c), yielding a calibration equation to convert pseudo-Thellier slopes into absolute intensity values. This linear relationship is given by: B_abs_ (μT)=7.371 × pseudo-Thellier slope+14.661. It is important to note that this relationship depends on the field strength used to impart the ARM during the pseudo-Thellier experiments (in this study 40 μT, see Methods). The non-zero *y* axis intercept must be explained by the relationship behaving nonlinearly towards (much) lower intensities (being, for example, a Langevin function). The high degree of agreement between the pseudo-Thellier results and the field models CALS3K.4 (refs 31, 32), CALS10K.1b^33^, ARCH3K.1 (refs 31, 32), GUFM1 (ref. 14) and IGRF (Fig. 1d) and other archaeointensity methods (Fig. 2) strongly supports that the linear approximation is appropriate for the range used in this study.

For each of our sites, four to ten samples were processed to produce 413 data points in total (Supplementary Table S6); 250 of these are younger than 1840 AD and can be used for calibration purposes. The 413 data points in the pseudo-Thellier data set are grouped by their age; if four or more samples per group pass the selection criterion, the pseudo-Thellier results of those samples are averaged and converted to absolute archaeointensities (Supplementary Table S7). Out of 33 possible age groups, we yield 20 reliable calibrated pseudo-Thellier archaeointensities, a success rate of ~61%; substantially higher than the ~27% success rate associated with our thermal archaeointensity experiments. Furthermore, we resolve the observed trend in the geomagnetic field intensity at Hawaii for the historical age range (Fig. 1d); all the 10 calibrated pseudo-Thellier estimates are within 3 μT of their expected values and randomly distributed about them. This gives confidence that the calibrated pseudo-Thellier palaeointensity approach can reliably be used also for older sites.

### Previously reported archaeointensities

Finally, we augmented our measurements with carefully selected data from previous archaeomagnetic studies on Hawaii as provided by the GEOMAGIA database^34^. We downloaded 107 intensity entries for the age range considered in this paper. First, we only considered intensity entries that have their own ^14^C dating associated with them; this rejects 42 entries. Second, directional information should be provided along with the intensity data so that virtual dipole moments (VDMs) can be calculated; this information is missing for another 27 entries. Third, we required an entry to be the mean of at least four independent results, with its s.d. being <20% of the mean. As different authors report different Thellier-style parameters, and select their most reliable results accordingly, it was not possible to reinterpret all data and subject them to the same set of selection criteria. Where authors had presented different ‘classes’ of intensity results, only the highest quality subset was taken. We accept 15 archaeointensity entries^9^,^10^,^11^,^12^,^27^ from the GEOMAGIA database after our careful selection procedure (Supplementary Fig. S4, Supplementary Table S8). Directional information was accepted if associated ^14^C ages were provided—a criterion met by 143 entries^35^ (Supplementary Table S9). Some of the ^14^C ages associated with either directional or archaeointensity results were not calibrated using a proper radiocarbon calibration curve or were calibrated using a curve that is by now superseded. The original dating-sample codes and ^14^C laboratory ages were specified in all reporting studies. We therefore recalibrated all specified ^14^C datings by means of the newest INTCAL.09 curve using the Calib 6.0 routine^18^,^19^ (Supplementary Table S9).

## Discussion

We compiled all accepted data into a full vector description of the regional behaviour of the geomagnetic field (Fig. 2). An intensity curve was computed based on maximum likelihoods, together with its one s.d. interval (Fig. 2e,f). The quality of our record is strengthened considerably through the observation that near-coeval archaeointensities obtained using different methods and from different cooling units agree within experimental uncertainty. Different lavas proved to be successful in certain archaeointensity techniques but failed in others; thus, it is important to apply various techniques within one archaeointensity study. Our calibrated pseudo-Thellier method proved to be an important addition to absolute archaeointensity methodologies, elucidating the geomagnetic field intensity for sites that fail in absolute methods. With our new multi-method approach, we were able to obtain reliable archaeointensity estimates for 22 out of the 33 possible ages. This constitutes an unusually high success rate of ~67%, further supporting the application of this approach to future studies aiming to construct a reliable regional full vector record from a volcanic edifice.

The obtained intensity curve shows a constant value of ~40 μT from 200 to ~850 AD. Between ~846 and 977 AD, the field intensity at Hawaii rose quickly from ~40 to ~63 μT. The following short period of very high field intensity up to ~1150 AD is robustly indicated by five results from four lava flows measured using three different techniques. Owing to Hawaii’s low latitude, the high intensity corresponds to a VDM peaking at ~140 ZAm^2^—75% higher than today’s average value. Between 1120 and 1170 AD, the archaeointensity rapidly decreased again and slowly thereafter, with a modest recovery suggested around 1650 (but note the limited age precision associated with this single result shown in Fig. 2). Although precise estimates of the rates of decrease and increase are hampered by the resolution of our data, average rates of change of at least 150–250 nT per year are associated with the observed intensity high, approximately one order of magnitude higher than today’s rate of decrease at Hawaii.

**Figure 3:**
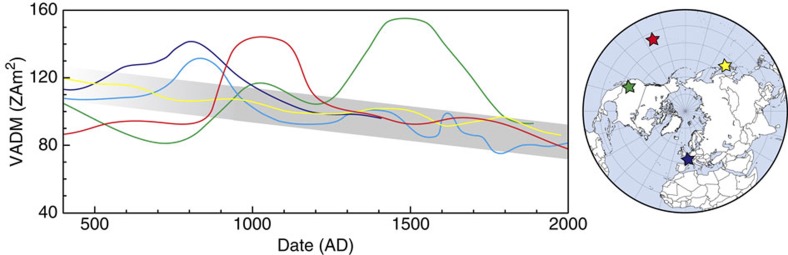
Intensity curves for four different regions. Western Europe^2^ (dark blue); France^1^ (light blue); Mesoamerica^4^ (green); Japan^36^ (yellow); and Hawaii (red). These locations are indicated by stars in the corresponding colour on the map of the northern hemisphere. The European and Mesoamerican curves are based primarily on thermal Thellier methods obtained from archaeological artefacts and show a peak in intensity in Europe around 800 AD and in Mesoamerica around 1500 AD. The Japanese curve is based on rigorously selected thermal Thellier experiments on lavas, showing a gradual decay in archaeointensity without short-lived intensity highs. The peak in intensity found for Europe predates the peak found for Hawaii by ~200 years, the peak in Mesoamerica post-dates the peak in Hawaii by ~450 years. The grey shaded area tentatively indicates a steadily decaying global geomagnetic field intensity (that is, dipole contribution) from which the short-lived regional intensity highs depart; the coherence between the curves decays before ~900 AD; the tentative trend is therefore faded for this period.

There are now reliable records of the variation in geomagnetic field intensity for Europe^1^,^2^, Mesoamerica^3^,^4^, Japan^36^ and Hawaii for the past 1,600 years; these records are reasonably well distributed over the northern hemisphere (Fig. 3). Apart from the occurrence of short-lived increases in the Hawaiian and Mesoamerican records, the reported virtual axial dipole moments for these four locations are notably similar between 1000 and 2000 AD. This implies a relatively homogenous field behaviour and very likely reflects the dipole decaying steadily from ~105 ZAm^2^ in 1000 AD to ~85 ZAm^2^ in 2000 AD—a rate of 2 ZAm^2^ per century. Immediately before 1000 AD, there was an intensity peak in Western Europe^1^,^2^ similar to those observed later in Hawaii and Mesoamerica. The four records show less coherence with one another in this earlier period. It is, however, currently unclear whether this is caused by reduced temporal resolution and therefore reliability in the virtual axial dipole moment curves or increased geomagnetic variability at smaller spatial scales. It is worth noting that this time period is coincident with a relatively reduced dipole component in the global field model CALS10K.1b^33^.

Overall, it appears that geomagnetic intensity behaviour over the last millennium can be decomposed into two components. The first is a long-term, nearly steady decay in the dipole component extending, backwards, the recent instrumental record-based trend^14^,^15^. The second is a series of rapid and severe fluctuations in intensity that our observations exclude as having a significant dipole contribution. They are neither globally synchronous nor exhibit opposite behaviour for locations that differ ~180° in longitude as expected for dipole tilt. It is furthermore unlikely that the observed rapid variations are caused by a single higher-order phenomenon and westward drift, as the peak in Hawaii predates that in Mesoamerica. We therefore conclude that the rapid fluctuations in the last 1,600 years must originate from intense, small-scale and potentially unrelated geomagnetic processes that occur and subside within tens to hundreds of years. Such spatially and temporally non-uniform features are, as yet, entirely enigmatic in terms of their physical cause but appear to be a fundamental trait of the behaviour of the geomagnetic field, and geodynamo operation, on centennial timescales.

## Methods

### Thermal IZZI-Thellier measurements

The thermal Thellier measurements were made on an AGICO JR-5 spinner magnetometer using cubic samples with sides of 10 mm and followed the IZZI-Thellier protocol^21^,^25^,^26^. The temperature steps are as follows (all in °C, Z=zero field, I=in field (35 μT), ZC=tail-check, IC=pTRM-check): 100 Z, 100 I, 150 I, 150 Z, 100 IC, 200 Z, 200 I, 200 ZC, 300 I, 200 IC, 350 Z, 350 I, 350 ZC, 400 I, 400 Z, 350 IC, 450 Z, 450 I, 450 ZC, 480, I, 480 Z, 480 IC, 500, I, 500 Z, 450 IC, 520 I, 520 Z, 480 IC, 530 I, 530 Z, 540 I, 540 Z, 520 IC, 550 I, 550 Z, 560 I, 560 Z, 540 IC, 570 I, 570 Z, 580 I and 580 Z. The program ThellierTool 4.2 (ref. 37) was used for data interpretation. To accept an archaeointensity estimate, at least four samples should pass the SELCRIT-1 selection criteria^38^,^39^ (Supplementary Table S3) and the mean should have a s.d. <20% of the mean. Generally, the lower temperature end (below 450 °C) of the Thellier experiments yielded the best results (Supplementary Table S4, Supplementary Fig. S1).

### Microwave Thellier measurements

Discs of 5 mm diameter and 2–3 mm thickness were measured using both 14 GHz microwave systems at the University of Liverpool^22^,^27^,^28^. The Coe^40^,^41^; IZZI^21^; and perpendicular^42^ protocols were applied to test the sensitivity of the results to the methodology. pTRM checks^43^ and pTRM tail checks^44^ were applied in some cases. Where the IZZI method or pTRM tail checks were used, the laboratory field was directed at an angle of at least 45° to the NRM so that multidomain effects were not made undetectable through sub-parallelism of the ancient and laboratory field vectors^45^. A laboratory field of 40 μT was applied in all experiments. As for the IZZI-Thellier experiments, a minimum of four results were required to produce an average required to have a s.d. <20% of the mean. The criteria used for the individual results are specified in Supplementary Table S3.

### Domain-state-corrected multispecimen method

For a meaningful MSP-DSC^23^,^24^ experiment, a large portion of the NRM, preferably at least 15%, should be unblocked at the temperature used in the experiment (referred to as set temperature); no or very small overprints should be present; and both chemical and magnetic alteration should be avoided. To test for these conditions, at least three samples per site are thermally demagnetized using an ASC-TH48 thermal demagnetizer and measured on a 2G DC-SQUID magnetometer (Supplementary Fig. S2, column a). Furthermore, cyclic low-field susceptibility-versus-temperature analyses were performed on an AGICO KLY-3S susceptometer with furnace attachment (Supplementary Fig. S2, column b). The peak temperatures of the six heating cycles are 220, 290, 360, 420, 500 and 600 °C; after each heating cycle, the temperature is lowered at least 50 °C to test the reversibility of the signal. The highest temperature reached in the last reversible segment is chosen as the ‘alteration temperature’ and is regarded as the highest temperature that can be used safely in thermal archaeointensity experiments.

On the basis of the alteration temperature and the percentage of NRM unblocked, one or more potential set temperatures are chosen per site. For these potential set temperatures, ARM tests^29^ are performed (Supplementary Fig. S2, column c). For 14 sites in this study that pass the ARM test, the MSP-DSC protocol^24^ was followed. A test for subtle alteration during the multiple heating steps is incorporated in this protocol; results are only accepted if the alteration during the experiment is ≤3%. Of the 14 MSP-DSC experiments, six passed this last selection criterion. The ‘α-parameter’^24^ is set to 0.5. To remove potential outliers from the MSP-DSC diagrams, the data points that fall outside the one-s.d. error envelope resulting from all measured data are removed, and the linear fit, together with the corresponding one-s.d. error envelope, is recalculated using only the accepted data points (Supplementary Fig. S2, column d; Supplementary Table S5).

### Pseudo-Thellier experiments

All pseudo-Thellier measurements are performed on a robotized 2G DC-SQUID magnetometer, with demagnetization coils attached to the system; AF fields higher than 100 mT are applied manually in a laboratory-built demagnetization coil. The pseudo-Thellier experiments consist of three steps. First, the NRM of the samples is demagnetized in 18 field steps (0, 2.5, 5, 7.5, 10, 15, 20, 25, 30, 40, 50, 60, 70, 80, 100, 150, 225 and 300 mT). After the demagnetization at 300 mT, the magnetization of the samples is generally reduced to a few per cent of their NRM. The NRM is demagnetized in three perpendicular axes, without aligning the NRM to either one of them. In the second step of the pseudo-Thellier protocol, a sequence of ARMs is imparted on the samples using the same AF field steps as in the NRM demagnetization. The ARM is imparted in the axial direction of the magnetometer system; as anisotropy in extrusive igneous rocks is generally <2%, the NRM and ARM can be reliably compared, even though they may not have the same direction. The DC bias field for the ARM acquisitions is set to 40 μT. The last step of our pseudo-Thellier protocol is to demagnetize the imparted ARMs, again using the same field steps.

To obtain reliable pseudo-Thellier slopes that compare the field in which the NRM was acquired among sites, the same grains that carry the NRM must carry the ARM in the samples. To assess the extent to which this is the case, the demagnetization experiments of the NRM and the ARM (step 1 and step 3) are plotted against each other for each field step (Supplementary Fig. S3, panel a). If the same grains carry the NRM and the ARM, it is to be expected that the demagnetization curves behave proportionally—that is, the fit through the plot of the two is linear. For small AF fields, below 10 mT, small overprints in the NRM or viscous behaviour sometimes causes the data points to deviate from the linear fit. The linear segment is usually between 15 and 100 mT, and these fields are subsequently used to obtain the pseudo-Thellier slope. The AF field segment used is specified per sample in Supplementary Table S6.

The pseudo-Thellier slope is obtained from an Arai plot of the NRM remaining (data from step 1) on the vertical axis against the ARM acquired (step 2) on the horizontal axis (Supplementary Fig. S3, panel b). The pseudo-Thellier slope is (the absolute) slope of the linear fit through the data points corresponding to the AF fields selected above. This slope is a measure of the intensity of the NRM-imparting field with respect to the ARM-imparting field. As the imparting DC bias field is equal for all samples in this experiment, the pseudo-Thellier slope is a relative indicator of the archaeointensity.

## Author contributions

L.V.dG. and M.J.D. designed the project and participated in the fieldwork. L.V.dG. performed the rock-magnetic, multispecimen and pseudo-Thellier experiments; E.H.-B. participated in the fieldwork and performed the thermal Thellier experiments; A.J.B. carried out the microwave study. L.V.dG., M.J.D. and A.J.B. interpreted all results and are the prime co-authors of the manuscript. C.G.L. contributed to the geomagnetic interpretation and the writing of the manuscript.

## Additional information

**How to cite this article:** de Groot, L. V. *et al.* Rapid regional perturbations to the recent global geomagnetic decay revealed by a new Hawaiian record. *Nat. Commun.* 4:2727 doi: 10.1038/ncomms3727 (2013).

## Supplementary Material

Supplementary Information Supplementary Figures S1-S4 and Supplementary Tables S1-S9
