# Supplementary material for: Rapid regional perturbations to the recent global geomagnetic decay revealed by a new Hawaiian record
Source: Nat Commun. 2013 Oct 31;4:2727. doi: 10.1038/ncomms3727 (PMC3826623; doi:10.1038/ncomms3727)
Supplement: Supplementary Information — Supplementary Figures S1-S4 and Supplementary Tables S1-S9 [file ncomms3727-s1.pdf]

Supplementary information

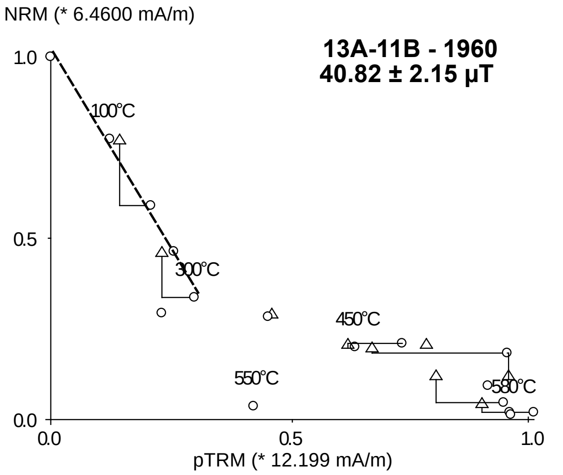

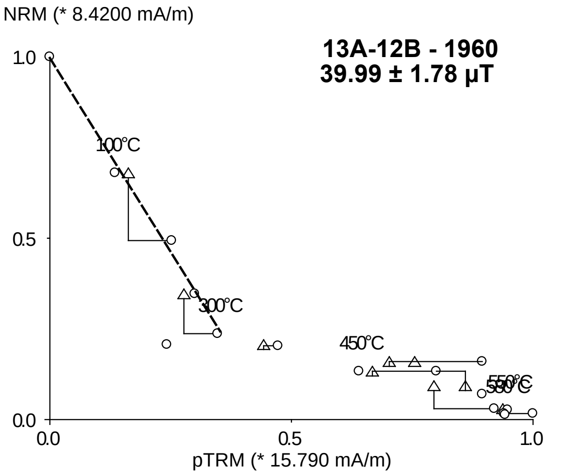

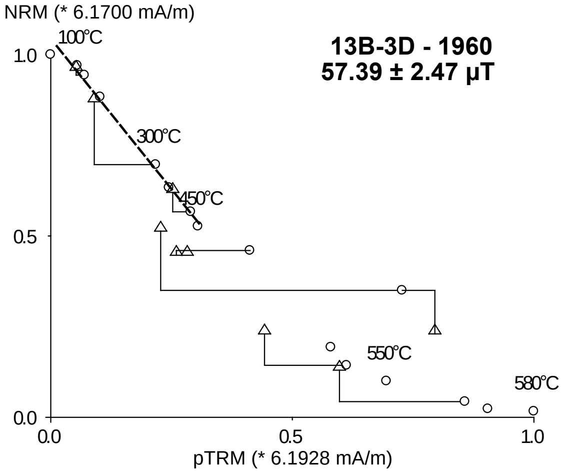

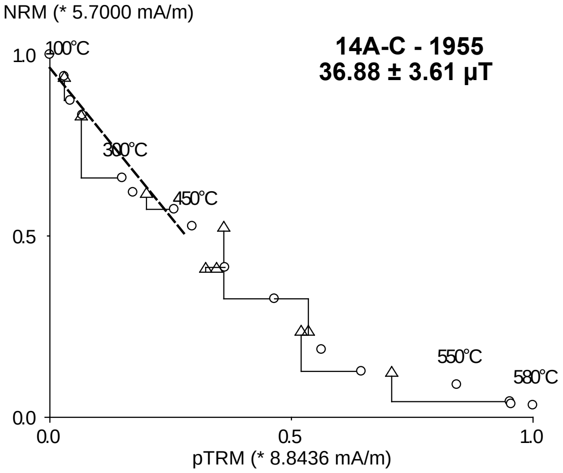

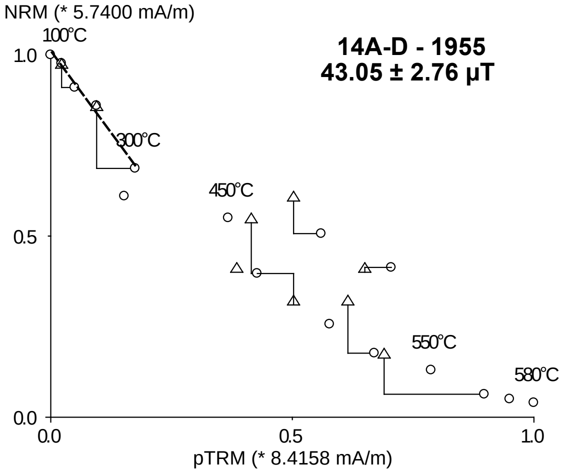

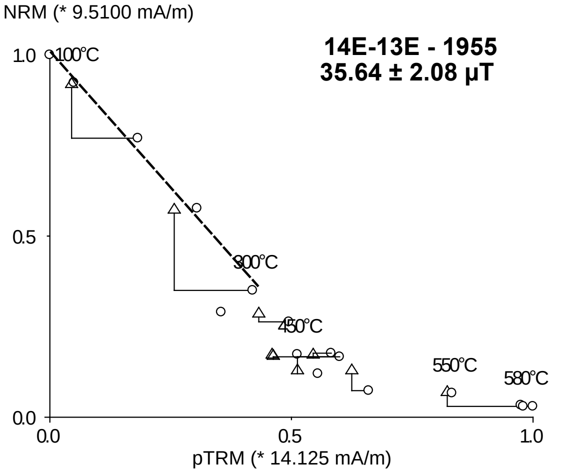

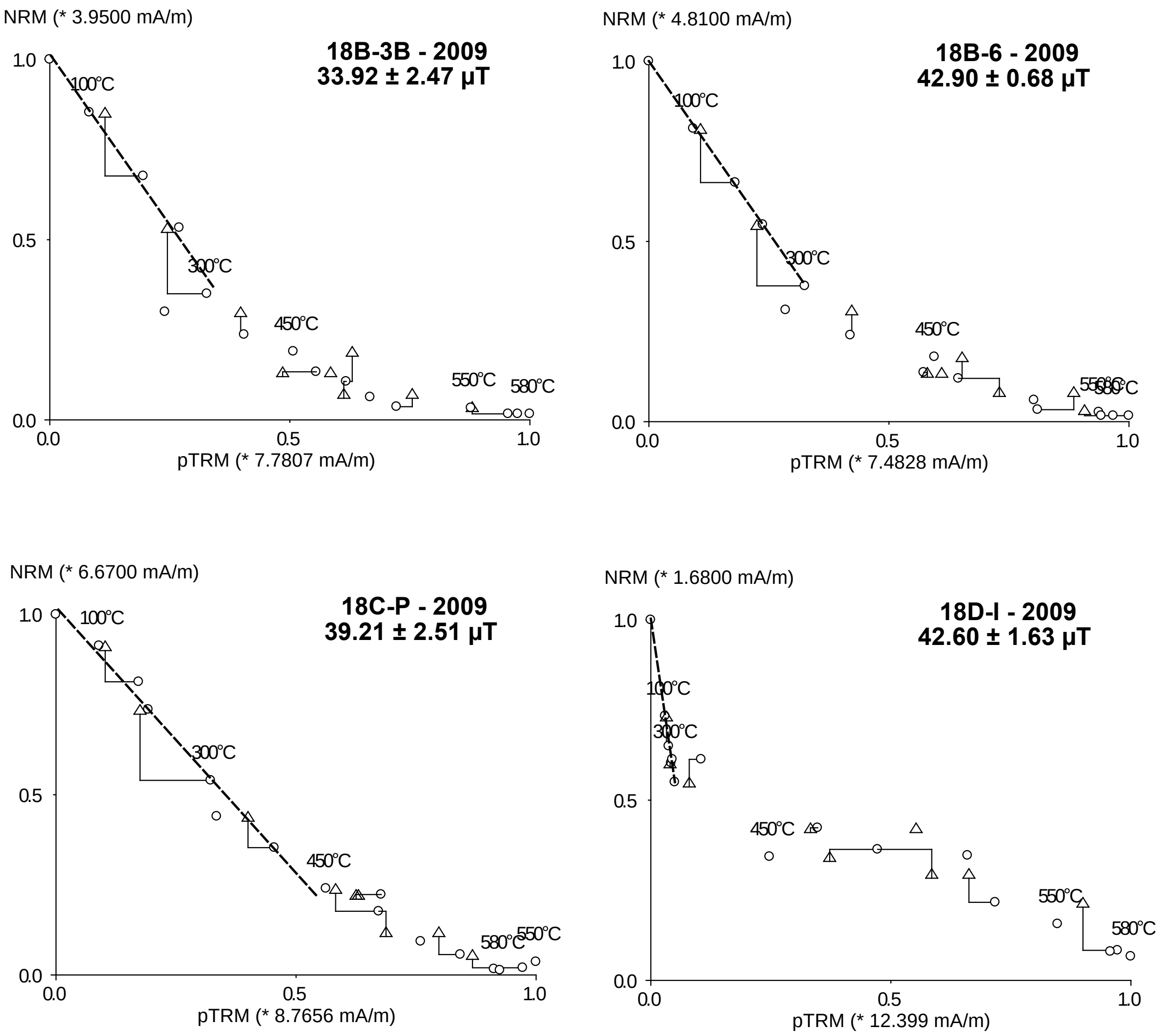

Supplementary Fig. S1 – Thermal IZZI-Thellier results.

For all accepted thermal Thellier results the Arai plots are provided. Sample names and the obtained archaeointensity are in the upper right corner of each panel. Data are plotted as open circles; checks are depicted as open triangles; the dashed lines are the interpreted linear regressions.

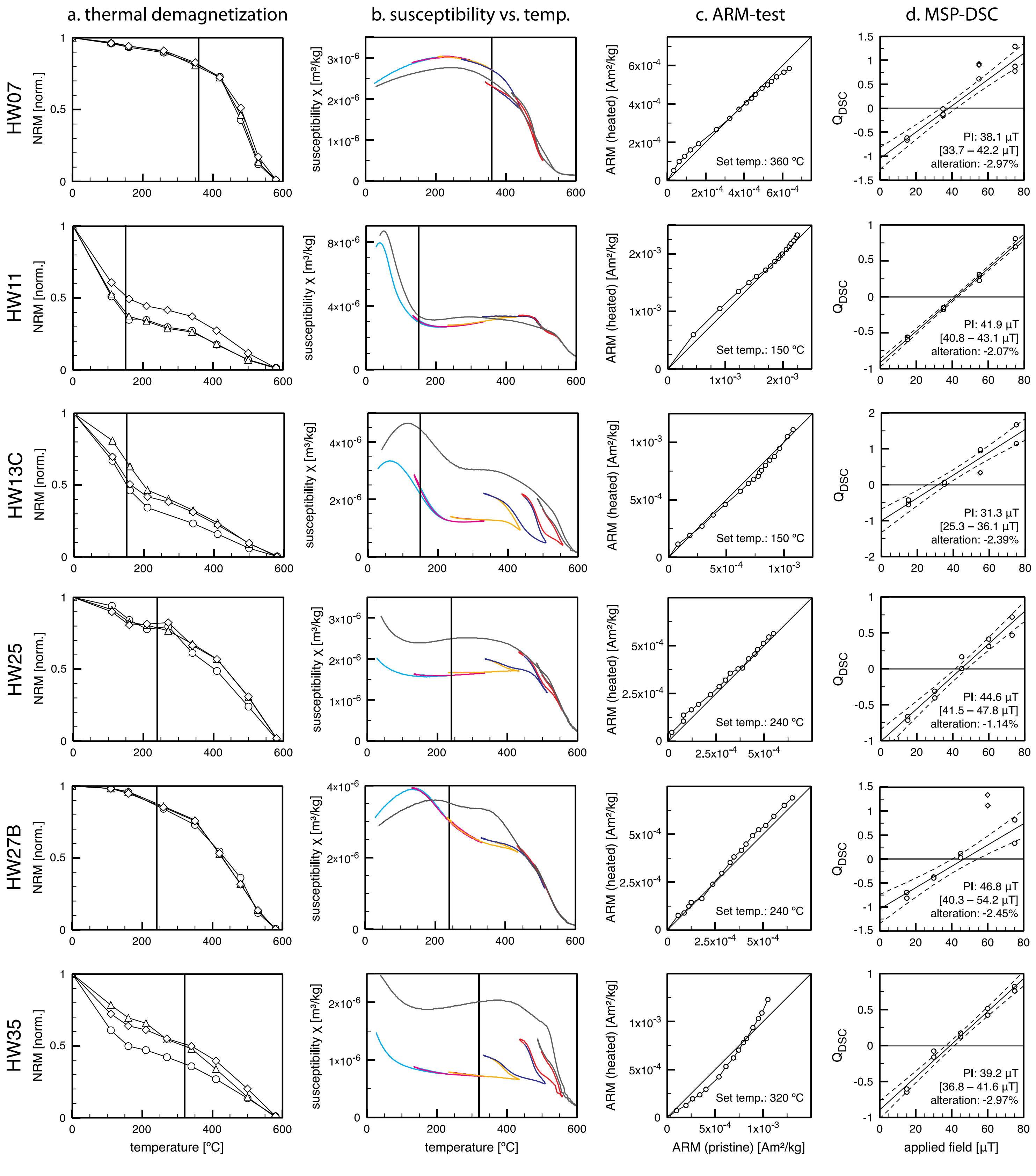

Supplementary Fig. S2 – Domain state corrected multispecimen archaeointensity results.
In column a are the thermal demagnetization results for all six samples that were successful in the MSP-DSC[22,23] experiments; per site three samples were analysed, each sample is depicted by different symbols: circles, triangles, or diamonds. The set temperature of the MSP-DSC experiments is indicated by a solid black vertical line, ideally a larger portion of the NRM should be unblocked at this temperature to ensure a steep fit in the MSP-DSC experiments. The results of the thermally cycled susceptibility-versus-temperature experiments are in column b. The temperature was increased in six thermal cycles with increasing peak temperatures; these cycles are indicated by different colours. The susceptibility should be reversible, since irreversible behaviour indicates alteration. Again the set temperature of the MSP-DSC experiment is indicated as a solid black vertical line. The ARM-tests[29] in column c show acceptable behaviour for these six sites: the data (open circles) plots on the diagonal within reasonable error. The results of the MSP-DSC experiments are in column d; circles depict the accepted data, rejected data are open diamonds. The linear regression is shown as the solid black line, together with its one standard deviation error envelope (dashed lines). The obtained archaeointensity and confidence interval are indicated in the lower right corner of each panel. Furthermore, the alteration between the first and the fourth step of the MSP-DSC protocol is indicated, results are rejected if this alteration is more than 3%.

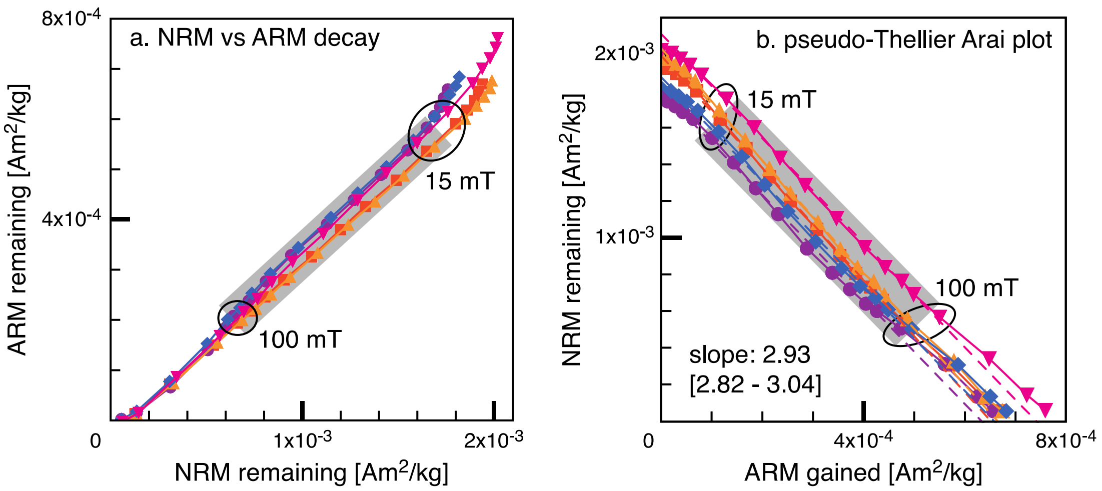

Supplementary Fig. S3 – An example of the pseudo-Thellier analyses.
In panel a the demagnetization curves of the natural remanent magnetization (NRM) and the anhysteretic remanent magnetization (ARM) are compared. The data of five samples are shown by five different colours and symbols (purple circles, blue diamonds, pink triangles, orange squares and yellow triangles). If they behave proportionally it is assumed that the same grains that carry the NRM carried the ARM. In this example, an AF field range from 15 to 100 mT is selected for the five separate samples from site 17B (grey shaded area). On the right (panel b) is the Arai plot of the pseudo-Thellier experiment for the same five samples, shown using the same colours and symbols as in panel a; the slopes of the five samples are interpreted from 15 to 100 mT. The average slope, together with its one standard deviation confidence interval is given in the lower left corner of the panel.

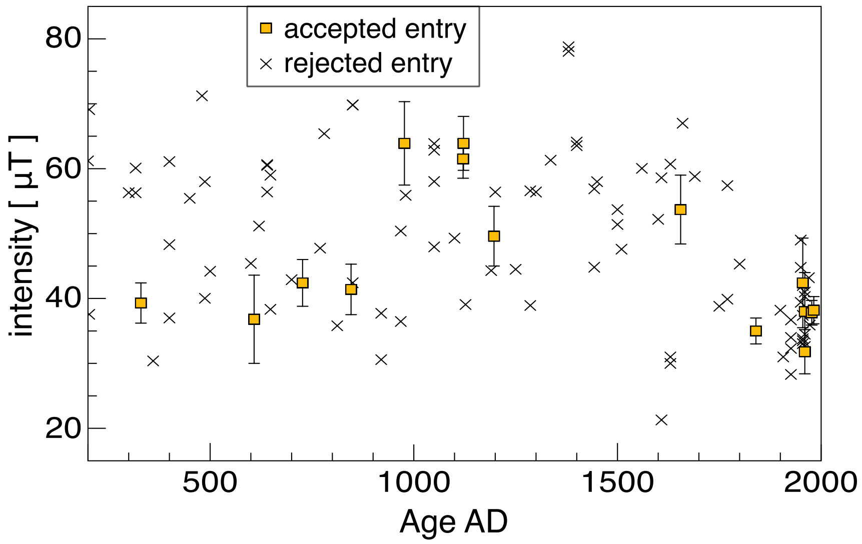

Supplementary Fig. S4 - Accepted GEOMAGIA entries.

Accepted GEOMAGIA entries are shown in yellow squares, together with their reported confidence interval (vertical error bar), rejected entries are shown as grey crosses. The ^{14}C-datings associated with the accepted GEOMAGIA entries were recalibrated using the INTCAL.09 curve; the archaeointensity results are plotted at their mean probability age AD.

Supplementary Table S1 – Site details for the historically dated lava flows.

From left to right: Site code (number refers to a geographic location, suffix A-E to sites usually taken at different levels in the lava flow at one location); the date (AD); latitude, longitude, elevation (Elev.) and associated GPS accuracy (GPS acc.) of the site; the thickness of the flow at that location ('<' indicates that the base of the flow is not visible); the level with respect to the top of the flow at which the samples were taken; and the IGRF reference details comprising the predicted declination, inclination and intensity.

site	age	latitude (N)	longitude (W)	Elev. [m]	GPS acc. [m]	flow thick-ness [cm]	depth from top [cm]	IGRF declination	IGRF inclination	IGRF intensity[nT]
**1**	1955	19° 26.168′	154° 56.703′	320	4	150	70	10° 56'	36° 58'	36,241.6
**2**	1955	19° 23.733′	154° 55.653′	25	3	80	40	10° 55'	36° 54'	36,235.8
**3**	1907	19° 4.315′	155° 44.314′	603	4	<270	20	9° 35'	36° 56'	37,545.5
**4**	1907	19° 5.419′	155° 47.382′	609	4	<185	85	9° 35'	36° 57'	37,546.1
**5**	1887	19° 4.103′	155° 43.704′	619	5	270	190	-	-	-
**6**	1868	19° 3.558′	155° 41.678′	631	5	180	100	-	-	-
**7**	1868	19° 3.515′	155° 40.874′	652	5	80	35	-	-	-
**12**	1960	19° 30.779′	154° 49.127′	35	5	108	0	10° 56'	37° 11'	36,157.2
**13A**	1960	19° 30.779′	154° 49.127′	35	5	108	10	10° 56'	37° 11'	36,157.2
**13B**	1960	19° 30.779′	154° 49.127′	35	5	108	55	10° 56'	37° 11'	36,157.2
**13C**	1960	19° 30.779′	154° 49.127′	35	5	108	100	10° 56'	36° 55'	36,237.7
**14A**	1955	19° 23.938′	154° 55.147′	30	3	200	15	10° 55'	36° 55'	36,237.7
**14B**	1955	19° 23.938′	154° 55.147′	30	3	200	50	10° 55'	36° 55'	36,237.7
**14C**	1955	19° 23.938′	154° 55.147′	30	3	200	85	10° 55'	36° 55'	36,237.7
**14D**	1955	19° 23.938′	154° 55.147′	30	3	200	130	10° 55'	36° 55'	36,237.7
**14E**	1955	19° 23.938′	154° 55.147′	30	3	200	180	10° 55'	36° 51'	35,298.0
**15A**	1990	19° 21.728′	154° 57.976′	27	4	<100	10	10° 27'	36° 51'	35,298.0
**15B**	1990	19° 21.728′	154° 57.976′	27	4	<100	55	10° 27'	37° 11'	36,152.9
**17A**	1960	19° 30.424′	154° 50.463′	45	5	<<400	-	10° 55'	37° 11'	36,152.9
**17B**	1960	19° 30.424′	154° 50.463′	45	5	<<400	-	10° 55'	37° 11'	36,152.9
**17C**	1960	19° 30.424′	154° 50.463′	45	5	<<400	-	10° 55'	35° 51'	34,696.8
**18A**	2009	19° 20.520′	155° 0.234′	52	5	<150	5	9° 44'	35° 51'	34,696.8
**18B**	2009	19° 20.520′	155° 0.234′	52	5	<150	40	9° 44'	35° 51'	34,696.8
**18C**	2009	19° 20.520′	155° 0.234′	52	5	<150	65	9° 44'	35° 51'	34,696.8
**18D**	2009	19° 20.520′	155° 0.234′	52	5	<150	150	9° 44'	37° 20'	36,522.5
**19A**	1935	19° 38.076′	155° 29.902′	2199	4	120	30	10° 36'	37° 20'	36,522.5
**19B**	1935	19° 38.076′	155° 29.902′	2199	4	120	65	10° 36'	37° 20'	36,522.5
**19C**	1935	19° 38.076′	155° 29.902′	2199	4	120	93	10° 36'	37° 20'	36,522.5
**20**	1790	19° 26.717′	154° 51.314′	16	6	25	15	-	-	-
**22**	1843	19° 37.996′	155° 30.527′	2211	4	50	30	-	-	-
**23**	1899	19° 38.195′	155° 29.049′			80	55	-	-	-
**24**	1942	19° 32.616′	155° 33.709′	3274	5	-	-	10° 48'	36° 58'	36,368.2
**32**	1855	19° 34.959′	155° 27.292′	2551	3	-	-	-		
**37**	1926	19° 11.512′	155° 51.938′	542	4	230	160	10° 15'	36° 30'	36,653.7
**38**	1919	19° 13.835′	155° 52.518′	499	4	50	42	10° 40'	36° 38'	36,937.9
**39**	1950	19° 15.990′	155° 52.506′	426	3	95	35	10° 54'	36° 39'	36,151.9
**40**	1800-	19° 46.221′	155° 55.817′	633	3	400	340	-	-	-
**41**	1859	19° 49.589′	155° 49.405′	683	3	150	125	-	-	-
**42**	1800-	19° 45.654′	155° 1.498′	68	3	120	55	-	-	-
**43**	1800-	19° 49.841′	155° 56.437′	104	5	185	95	-	-	-

Supplementary Table S2 – Site details for all ^{14}C-dated sites.

From left to right: the site code (numbers refer to geographic locations, characters A or B if more than one site taken at the same location); reference to the dating study; the laboratory ^{14}C age (before present), with associated laboratory ^{14}C error; the median probable age after recalibration using INTCAL.09[17,18], together with the one standard deviation intervals as obtained from the recalibration; and the latitude, longitude, elevation and associated GPS accuracy at which the samples were taken.

Site	Dating study ref.	dating sample no.	^{14}C age [BP]	^{14}C error [years]	Median prob. age [AD]	One standard deviation intervals (lower limit – upper limit; probability)			latitude (N)	longitude (W)	Elev. [m]	GPS acc. [m]
**8**	15	W5048	340	60	1554	1484 - 1529; 0.328	1543 - 1634; 0.672		19° 28.894'	154° 51.782'	124	7
**9**	16	W5631	580	200	1380	1218 - 1522; 0.915	1574 - 1584; 0.016	1590 - 1625; 0.069	19° 29.940'	155° 7.528'	613	5
**10**	15	W4981	1470	50	588	558 - 640; 1.000			19° 38.178'	155° 2.963'	134	11
**11**	15	W4343	1280	70	747	659 - 781; 0.907	790 - 809; 0.093		19° 39.832'	155° 7.113'	263	4
**16**	15	W5325	1370	200	670	436 - 489; 0.098	512 - 516; 0.007	530 - 887; 0.894	19° 13.495'	155° 27.650'	400	4
**21**	15	W4688	490	60	1424	1327 - 1342; 0.099	1394 - 1458; 0.901		19° 27.360'	154° 55.532'	275	3
**25**	15	W3860	670	60	1323	1275 - 1318; 0.537	1352 - 1390; 0.463		19° 13.767'	155° 27.393'	418	5
**26**	15	W5211	960	70	1090	1020 - 1156; 1.000			19° 11.384'	155° 27.273'	100	12
**27A**	15	W4137	890	60	1135	1045 - 1094; 0.353	1120 - 1141; 0.147	1147 - 1214; 0.501	19° 8.322'	155° 33.119'	390	4
**27B**	15	W4137	890	60	1135	1045 - 1094; 0.353	1120 - 1141; 0.147	1147 - 1214; 0.501	19° 8.323'	155° 33.113'	413	6
**28**	15	W4631	1110	60	924	881 - 998; 0.955	1003 - 1013; 0.045		19° 42.143'	155° 5.031'	58	7
**29**	15	W4404	530	70	1394	1316 - 1354; 0.372	1389 - 1442; 0.628		19° 12.611'	155° 27.525'	293	4
**33**	16	W5985	1580	120	463	349 - 369; 0.060	378 - 603; 0.940		19° 41.100'	155° 27.980'	2030	6
**34**	15	W4012	740	60	1264	1219 - 1295; 1.000			19° 8.845'	155° 33.772'	502	4
**35**	16	W5757	410	150	1530	1329 - 1340; 0.024	1396 - 1655; 0.976		19° 20.853'	155° 23.375'	878	4
**36**	16	W5985	1580	120	463	349 - 369; 0.060	378 - 603; 0.940		19° 41.100'	155° 27.980'	2030	6
**45**	15	W5633	1600	200	425	239 - 645; 1.000			19° 32.363'	155° 48.495'	1465	3

Supplementary Table S3 – Selection criteria for Thellier results.
The selection criteria as applied to the IZZI (thermal) Thellier results (middle column) and the microwave Thellier results (rightmost column).

	Thermal (SELCRIT1)	Microwave
**Number of measurements included (n)**	> 4	> 4
**Fraction of NRM lost during the experiment (f)**	≥ 0.15	≥ 0.3
**Quality factor[40]**	≥ 1	≥ 4
**The angle between the origin-anchored and floating line fits in the Zijderveld plot (α)**	≤ 0.15	-
**The ratio of the standard deviation of the mean and the mean (β)**	≤ 0.1	≤ 0.1
**Mean angular deviation (MAD, free)**	≤ 15°	-
**DRAT[37]**	≤ 10	-
**CK-error[46]**	≤ 10	-
**The discrepancy of a pTRM check normalized by the original NRM (Δ pTRM)**	-	≤ 10%
**The sum of the signed values of Δ pTRM (Σ (Δ pTRM))**	-	≤ 20%

Supplementary Table S4 – Thermal Thellier results.

From left to right: sample code; obtained archaeointensity (int.) with its standard deviation (st.dev.); Temperature segment in the Thellier experiment (T); number of data points used for the linear fit (N); fraction of the NRM unblocked (f); gap-factor (g); quality factor (q); alpha (α); the ratio of the standard deviation and the obtained archaeointensity (β); the mean angular deviation (MAD); DRAT; and the CK-error. The selection criteria used are on the second line in the table. The data for 1955 and 1960 as well as for 2009 are averaged.

sample	int.	st.dev.	T	N	f	g	q	α	β	MAD free	Drat	CK-error
**SELCRIT:**				**> 4**	**≥ 0.15**		**≥ 1**	**≤ 15**	**≤ 0.1**	**≤ 15**	**≤ 10**	**≤ 10**
				**1955 & 1960 AD (thermal Thellier)**								
**13A-11B**	40.8	2.15	20 - 300	5	0.65	0.72	8.9	9.3	0.05	8.3	4.9	4.9
**13A-12B**	40.0	1.78	20 - 300	5	0.76	0.71	12.1	3.8	0.04	5.9	5.2	6
**13B-3D**	57.4	2.47	20 - 450	8	0.47	0.78	8.5	3.5	0.04	8.3	1.8	1.7
**14A-C**	36.9	3.61	20 - 450	8	0.48	0.80	3.9	6.1	0.10	9.7	7.1	4.9
**14A-D**	43.1	2.76	20 - 300	5	0.31	0.66	3.2	1.0	0.06	4.7	0.2	0.1
**14E-13E**	35.6	2.08	20 - 300	5	0.64	0.73	7.9	0.5	0.06	7.1	7.7	7
**n = 6**	**42.3**	**7.9 (19%)**										
				**2009 AD (thermal Thellier)**								
**18B-3B**	33.9	2.47	20 - 300	5	0.63	0.75	6.5	6.1	0.07	10.3	7.1	6.2
**18B-6**	42.9	0.68	20 - 300	5	0.62	0.74	29.3	11.5	0.02	13.6	2.9	2.9
**18C-P**	39.2	2.51	20 - 450	8	0.78	0.83	10.1	6.5	0.06	6.3	8.2	9.6
**18D-I**	42.6	1.63	20 - 350	6	0.45	0.58	6.9	4.5	0.04	9.7	4.3	3.1
**n = 4**	**39.7**	**4.2 (11%)**										

Supplementary Table S5 – Summary of MSP-DSC results.

For each successful experiment the following parameters are given: the temperature used in the experiment (set T); the obtained archaeointensity (Intensity); the one standard deviation confidence interval; the number of samples used in the experiment (the number of rejected sampled between brackets); the slope of the linear fit (a); the y-axis intercept of the linear fit (b); the coefficient of determination (r^2) and the change between steps m1 and m4 in the experiment expressed as a percentage of m1 (samples with more than 3% change were rejected). Results marked with * were published before[29].

Site	Set T	Intensity [μT]	Confidence interval [μT]	n (rejected)	a	b	r^2	Change m1 – m4
**07**	360	38.1	33.7 – 42.2	12 (2)	0.0274	-1.0439	0.959	-2.97%
**11**	150	41.9	40.8 – 43.1	12	0.0218	-0.9153	0.995	-2.07%
**13C**	150	31.3	25.3 – 36.1	12 (1)	0.0318	-0.9945	0.923	-2.39%
**14B***	250	35.9	30.2 – 42.0	12	0.0266	-0.9535	0.901	2.60%
**17A***	300	38.2	27.6 – 47.1	5.5	0.0312	-1.1948	0.862	-2.53%
**25**	240	44.6	41.5 – 47.8	10	0.0226	-1.0098	0.967	-1.14%
**27B**	240	46.8	40.3 – 54.2	10 (2)	0.0222	-1.0402	0.922	-2.45%
**35**	320	39.2	36.8 – 41.6	10	0.0228	-0.8947	0.983	-2.97%

Supplementary Table S6 – pseudo-Thellier results.

For every pseudo-Thellier result the following parameters are given: date AD; site code; reference intensity (if applicable, GUFM1 between 1840 and 1900 AD, IGRF after 1900 AD); obtained pseudo-Thellier slope; associated $B_{½ARM}$; the AF segment that defines the pseudo-Thellier slope; and the associated r^2 of this pseudo-Thellier slope. The table is sorted by date AD and within each age group by $B_{½ARM}$. The $B_{½ARM}$s passing the selection criterion are in black; the rejected ones are in red. If four or more samples per age group pass the selection criterion, an average is calculated (below the solid line), with its associated standard deviation (below the average).

date AD	site	reference intensity	pseudo-Thellier slope	$B_{½ARM}$ [mT]	AF segment	r^2
**2009**	18B	34.7	2.684	10.9	15 - 100	0.999
**2009**	18D	34.7	1.675	11.1	20 - 100	0.994
**2009**	18B	34.7	2.585	11.2	15 - 100	0.994
**2009**	18B	34.7	2.850	11.4	15 - 100	0.994
**2009**	18B	34.7	2.692	11.6	20 - 100	1.000
**2009**	18B	34.7	2.809	12.1	15 - 100	0.992
**2009**	18D	34.7	1.675	12.3	20 - 100	1.000
**2009**	18D	34.7	1.464	13.2	20 - 100	0.999
**2009**	18B	34.7	3.159	13.4	20 - 100	0.997
**2009**	18D	34.7	1.719	13.6	20 - 100	0.997
**2009**	18B	34.7	3.365	14.0	20 - 100	0.996
**2009**	18A	34.7	2.261	14.1	20 - 100	0.999
**2009**	18A	34.7	2.211	14.5	20 - 100	0.997
**2009**	18A	34.7	2.221	14.8	20 - 100	0.997
**2009**	18B	34.7	3.670	14.9	20 - 100	0.995
**2009**	18A	34.7	2.073	15.2	20 - 100	0.997
**2009**	18C	34.7	2.958	16.0	20 - 100	0.994
**2009**	18C	34.7	3.110	16.5	20 - 100	0.990
**2009**	18C	34.7	3.089	16.9	20 - 100	0.998
**2009**	18B	34.7	3.835	17.0	15 - 100	0.999
**2009**	18C	34.7	3.088	17.6	20 - 100	0.996
**2009**	18B	34.7	4.134	17.6	20 - 100	0.996
**2009**	18A	34.7	2.099	19.5	20 - 100	1.000
**2009**	18C	34.7	3.633	26.7	20 - 100	1.000
**2009**	**18**	**34.7**	**n < 4**			
**1990**	15B	35.3	2.121	21.8	15 - 100	0.995
**1990**	15B	35.3	2.224	22.9	15 - 100	0.999
**1990**	15B	35.3	2.327	25.2	7.5 - 100	0.996
**1990**	15B	35.3	2.383	25.6	7.5 - 100	0.999
**1990**	15B	35.3	3.713	26.5	7.5 - 100	0.998
**1990**	15A	35.3	2.782	27.2	7.5 - 100	0.999
**1990**	15B	35.3	3.032	29.1	15 - 100	0.997
**1990**	15B	35.3	2.700	31.4	15 - 100	0.999
**1990**	15A	35.3	2.309	32.3	0 - 100	0.999
**1990**	15A	35.3	2.289	34.3	0 - 100	1.000
**1990**	15A	35.3	2.433	37.5	7.5 - 100	0.994
**1990**	15A	35.3	2.316	38.5	0 - 100	0.994
**1990**	15A	35.3	2.494	41.2	7.5 - 100	0.999
**1990**	15A	35.3	2.027	46.8	7.5 - 100	0.998
**1990**	**15**	**35.3**	**2.567**			**0.998**
			**0.448**			
**1960**	13C	36.1	1.955	13.3	20 - 100	0.995
**1960**	12	36.1	2.699	14.8	20 - 80	0.996
**1960**	13A	36.1	2.274	15.4	20 - 100	0.999
**1960**	12	36.1	2.549	15.5	20 - 80	0.988
**1960**	12	36.1	2.570	15.9	20 - 80	0.992

**1960**	13A	36.1	2.130	16.2	20 - 100	0.997
**1960**	13A	36.1	2.250	16.8	20 - 100	0.999
**1960**	13C	36.1	1.885	16.9	20 - 100	0.988
**1960**	13C	36.1	2.190	17.2	20 - 100	0.990
**1960**	13A	36.1	2.308	17.3	20 - 100	0.996
**1960**	12	36.1	2.411	19.5	20 - 80	0.993
**1960**	12	36.1	2.566	19.7	20 - 80	0.991
**1960**	13C	36.1	2.148	21.7	20 - 100	0.996
**1960**	17C	36.1	3.186	27.1	20 - 100	0.994
**1960**	17C	36.1	3.538	27.7	20 - 100	0.995
**1960**	17C	36.1	2.802	36.1	20 - 100	0.995
**1960**	17C	36.1	3.253	36.7	20 - 100	0.998
**1960**	13B	36.1	2.939	38.3	7.5 - 80	0.998
**1960**	13B	36.1	3.033	38.9	7.5 - 80	0.988
**1960**	13B	36.1	2.865	39.3	20 - 80	0.993
**1960**	12	36.1	2.602	39.6	20 - 80	0.999
**1960**	17C	36.1	3.124	41.2	20 - 100	0.989
**1960**	13B	36.1	3.298	41.8	7.5 - 80	0.992
**1960**	17B	36.1	3.053	45.8	15 - 100	0.999
**1960**	17B	36.1	2.844	45.9	15 - 100	0.998
**1960**	17B	36.1	3.056	46.2	15 - 100	0.999
**1960**	17B	36.1	2.847	47.0	15 - 100	0.995
**1960**	17B	36.1	2.848	47.9	15 - 100	0.993
**1960**	17B	36.1	2.961	49.6	15 - 100	0.999
**1960**	17B	36.1	2.818	49.8	15 - 100	0.997
**1960**	17B	36.1	2.840	51.5	15 - 100	0.999
**1960**	12	36.1	2.989	51.5	20 - 80	0.998
**1960**	17B	36.1	3.066	51.8	15 - 100	1.000
**1960**	17B	36.1	3.078	52.4	15 - 100	0.996
**1960**	13B	36.1	3.628	59.1	20 - 80	0.989
**1960**	12	36.1	3.089	61.8	20 - 80	0.999
**1960**	13B	36.1	4.392	64.0	7.5 - 80	0.998
**1960**	13B	36.1	5.183	66.7	20 - 80	0.996
**1960**	13B	36.1	4.414	68.8	7.5 - 80	0.986
**1960**	13B	36.1	5.239	69.3	20 - 80	0.989
**1960**	13B	36.1	5.908	71.0	20 - 80	0.994
**1960**	12	36.1	3.140	91.7	20 - 80	0.997
**1960**	**multiple**	**36.1**	**3.033**			**0.996**
			**0.237**			
**1955**	2	36.2	1.142	13.1	20 - 100	0.997
**1955**	2	36.2	0.860	13.4	20 - 100	1.000
**1955**	2	36.2	0.915	13.8	20 - 100	0.999
**1955**	2	36.2	0.655	13.9	20 - 100	0.996
**1955**	1	36.2	1.345	15.3	10 - 100	0.996
**1955**	2	36.2	0.888	16.2	20 - 100	0.998
**1955**	14A	36.2	2.600	18.0	25 - 100	0.999
**1955**	1	36.2	1.453	18.0	10 - 100	0.990
**1955**	1	36.2	1.698	18.8	10 - 100	0.992
**1955**	1	36.2	1.367	19.5	10 - 100	0.993
**1955**	1	36.2	1.964	19.5	10 - 100	0.988
**1955**	14E	36.2	1.456	22.2	7.5 - 100	0.996
**1955**	14E	36.2	1.457	22.8	7.5 - 100	0.995
**1955**	14A	36.2	2.357	23.5	25 - 100	0.999
**1955**	14E	36.2	1.950	24.6	7.5 - 100	0.991
**1955**	14B	36.2	3.093	26.2	0 - 150	0.987
**1955**	14E	36.2	1.733	27.5	7.5 - 100	0.984
**1955**	14B	36.2	3.033	28.2	0 - 100	0.992
**1955**	14D	36.2	3.407	28.3	7.5 - 100	0.996

**1955**	14B	36.2	2.888	28.4	0 - 100	0.998
**1955**	14D	36.2	3.272	28.5	7.5 - 100	0.997
**1955**	14D	36.2	3.266	29.1	7.5 - 100	0.984
**1955**	14B	36.2	3.354	30.1	0 - 100	0.992
**1955**	14B	36.2	3.311	30.1	0 - 100	0.991
**1955**	14B	36.2	3.410	30.3	0 - 150	0.997
**1955**	14B	36.2	3.376	30.5	0 - 150	0.990
**1955**	14D	36.2	3.135	30.6	7.5 - 100	0.991
**1955**	14C	36.2	3.273	31.3	5 - 150	0.996
**1955**	14C	36.2	3.090	31.4	5 - 150	0.989
**1955**	14B	36.2	3.144	31.4	0 - 100	0.998
**1955**	14A	36.2	2.242	31.5	25 - 100	0.998
**1955**	14B	36.2	3.474	31.7	0 - 150	0.998
**1955**	14D	36.2	3.176	31.8	7.5 - 100	0.988
**1955**	14C	36.2	3.248	32.0	5 - 150	0.997
**1955**	14C	36.2	3.372	32.4	5 - 150	0.989
**1955**	14A	36.2	3.596	32.7	25 - 100	0.993
**1955**	14A	36.2	2.402	33.0	25 - 100	0.999
**1955**	14C	36.2	3.784	33.6	5 - 150	0.984
**1955**	**multiple**	**36.2**	**3.056**			**0.993**
			**0.516**			
**1950**	39	36.2	3.056	23.9	7.5 - 80	0.991
**1950**	39	36.2	2.876	24.0	7.5 - 80	0.989
**1950**	39	36.2	2.944	24.0	7.5 - 80	0.995
**1950**	39	36.2	2.940	24.1	7.5 - 80	0.997
**1950**	39	36.2	2.991	25.5	15 - 70	0.997
**1950**	39	36.2	3.148	25.9	15 - 70	0.983
**1950**	39	36.2	3.190	25.9	7.5 - 80	0.985
**1950**	39	36.2	3.028	26.2	15 - 70	0.991
**1950**	39	36.2	2.862	26.2	15 - 70	0.998
**1950**	39	36.2	3.192	27.1	15 - 70	0.986
**1950**	**39**	**36.2**	**3.023**			**0.991**
			**0.122**			
**1942**	24	36.4	3.120	7.7	15 - 100	1.000
**1942**	24	36.4	2.089	9.2	15 - 100	0.997
**1942**	24	36.4	2.503	9.5	15 - 100	1.000
**1942**	24	36.4	1.934	10.2	15 - 100	0.999
**1942**	24	36.4	1.871	10.7	15 - 100	0.994
**1942**	**24**	**36.4**	**n < 4**			
**1935**	19A	36.5	3.326	17.1	15 - 100	0.995
**1935**	19B	36.5	3.021	17.9	15 - 100	0.973
**1935**	19A	36.5	3.453	18.1	15 - 100	0.983
**1935**	19A	36.5	3.417	18.1	15 - 100	0.984
**1935**	19B	36.5	3.100	18.2	15 - 100	0.983
**1935**	19A	36.5	3.530	18.4	15 - 100	0.996
**1935**	19C	36.5	2.899	19.0	5 - 60	0.982
**1935**	19B	36.5	2.990	21.6	15 - 100	0.971
**1935**	19B	36.5	2.917	22.7	15 - 100	0.987
**1935**	19B	36.5	2.829	22.7	15 - 100	0.993
**1935**	19B	36.5	2.876	23.3	15 - 100	0.984
**1935**	19B	36.5	2.801	23.6	15 - 100	0.984
**1935**	19C	36.5	2.455	24.0	5 - 60	0.987
**1935**	19C	36.5	2.457	24.0	5 - 60	0.981
**1935**	19C	36.5	2.552	26.2	5 - 60	0.988
**1935**	19B	36.5	3.101	27.5	15 - 100	0.989
**1935**	19B	36.5	2.687	27.7	15 - 100	0.976

**1935**	19B	36.5	2.674	28.4	15 - 100	0.989
**1935**	**19**	**36.5**	**2.700**			**0.985**
			**0.222**			
**1926**	37	36.8	2.896	25.1	7.5 - 80	0.985
**1926**	37	36.8	2.817	26.6	7.5 - 80	0.996
**1926**	37	36.8	2.842	26.6	7.5 - 80	0.990
**1926**	37	36.8	2.846	27.6	7.5 - 80	0.991
**1926**	37	36.8	2.866	28.0	7.5 - 80	0.996
**1926**	37	36.8	2.944	28.5	15 - 70	0.992
**1926**	37	36.8	2.970	28.7	15 - 70	0.992
**1926**	37	36.8	2.922	30.3	15 - 70	0.990
**1926**	37	36.8	2.918	30.6	15 - 70	0.994
**1926**	37	36.8	2.989	33.2	15 - 70	0.994
**1926**	**37**	**36.8**	**2.901**			**0.992**
			**0.058**			
**1919**	38	37.1	0.612	9.3	15 - 70	1.000
**1919**	38	37.1	2.905	12.1	7.5 - 80	0.999
**1919**	38	37.1	1.947	14.3	15 - 70	0.999
**1919**	38	37.1	3.713	14.5	15 - 70	1.000
**1919**	38	37.1	3.450	14.5	15 - 70	0.999
**1919**	38	37.1	4.369	14.6	7.5 - 80	0.993
**1919**	38	37.1	3.601	15.0	7.5 - 80	0.993
**1919**	38	37.1	1.475	15.3	15 - 70	0.999
**1919**	38	37.1	3.179	16.5	15 - 70	0.994
**1919**	38	37.1	1.471	17.0	15 - 70	0.999
**1919**	38	37.1	3.796	17.5	15 - 70	0.999
**1919**	38	37.1	2.707	21.0	7.5 - 80	0.989
**1919**	38	37.1	2.798	25.6	15 - 70	0.990
**1919**	38	37.1	2.844	23.8	7.5 - 80	0.997
**1919**	38	37.1	2.749	24.3	15 - 70	0.986
**1919**	38	37.1	2.871	24.8	15 - 70	0.993
**1919**	**38**	**37.1**	**2.826**			**0.992**
			**0.054**			
**1907**	3	37.7	2.670	12.2	15 - 80	0.991
**1907**	3	37.7	2.180	18.0	15 - 80	0.961
**1907**	3	37.7	2.052	19.4	15 - 80	0.996
**1907**	3	37.7	2.003	19.5	10 - 50	0.980
**1907**	3	37.7	2.323	19.6	15 - 80	0.994
**1907**	3	37.7	2.289	19.9	15 - 80	0.984
**1907**	3	37.7	2.446	21.8	10 - 50	0.979
**1907**	3	37.7	2.293	22.0	10 - 50	0.982
**1907**	3	37.7	2.528	22.7	10 - 50	0.981
**1907**	3	37.7	2.743	22.9	10 - 50	0.960
**1907**	4	37.7	0.726	10.9	15 - 80	0.980
**1907**	4	37.7	0.643	11.1	15 - 80	0.937
**1907**	4	37.7	1.390	11.4	15 - 80	0.980
**1907**	4	37.7	1.271	11.7	15 - 80	0.984
**1907**	4	37.7	1.580	14.0	15 - 80	0.987
**1907**	4	37.7	0.252	14.5	20 - 100	0.614
**1907**	4	37.7	0.619	15.0	20 - 100	0.975
**1907**	4	37.7	0.741	15.6	20 - 100	0.980
**1907**	4	37.7	0.224	15.9	20 - 100	0.636
**1907**	4	37.7	0.307	16.7	20 - 100	0.763
**1907**	**multiple**	**37.7**	**n < 4**			
**1899**	23		1.658	13.1	15 - 100	0.979
**1899**	23		2.727	14.6	15 - 100	0.988

**1899**	23	1.995	15.0	15 - 100	0.977
**1899**	23	2.951	15.0	15 - 100	0.997
**1899**	**23**	**n < 4**			
**1887**	5	2.629	14.7	15 - 80	0.985
**1887**	5	2.646	15.8	15 - 80	0.978
**1887**	5	2.592	16.4	15 - 80	0.991
**1887**	5	2.428	18.0	15 - 80	0.985
**1887**	5	2.803	20.0	10 - 60	0.971
**1887**	5	2.849	21.3	10 - 60	0.972
**1887**	5	2.548	21.6	15 - 80	0.998
**1887**	5	2.782	22.8	10 - 60	0.979
**1887**	5	2.676	24.8	10 - 60	0.992
**1887**	5	2.784	25.4	10 - 60	0.985
**1887**	**5**	**n < 4**			
**1868**	6	3.260	21.8	15 - 80	0.997
**1868**	6	3.071	22.1	15 - 80	0.986
**1868**	6	3.484	23.3	20 - 70	0.996
**1868**	6	3.182	23.8	15 - 80	0.988
**1868**	6	3.377	24.3	20 - 70	0.996
**1868**	6	2.749	24.5	20 - 70	0.985
**1868**	7	3.137	24.9	15 - 80	0.998
**1868**	6	3.213	25.8	15 - 80	0.991
**1868**	6	2.717	25.8	20 - 70	0.989
**1868**	6	3.550	26.0	15 - 80	0.994
**1868**	6	2.946	29.0	20 - 70	0.982
**1868**	7	3.949	31.5	15 - 80	0.983
**1868**	7	3.925	33.9	15 - 80	0.979
**1868**	7	3.696	36.1	15 - 80	0.998
**1868**	7	3.015	36.3	15 - 80	0.995
**1868**	7	3.765	36.6	15 - 80	0.972
**1868**	7	3.245	37.8	15 - 80	0.988
**1868**	7	3.083	39.1	15 - 80	0.995
**1868**	7	3.661	39.5	15 - 80	0.992
**1868**	7	3.184	40.3	15 - 80	0.996
**1868**	**multiple**	**3.327**			**0.990**
		**0.373**			
**1859**	41	2.061	4.8	7.5 - 80	0.991
**1859**	41	2.219	4.8	7.5 - 80	0.995
**1859**	41	2.311	4.9	7.5 - 80	0.997
**1859**	41	2.258	4.9	7.5 - 80	0.996
**1859**	41	2.686	5.9	15 - 70	1.000
**1859**	41	2.941	6.1	15 - 70	0.996
**1859**	41	2.710	6.1	15 - 70	0.999
**1859**	41	3.333	6.5	15 - 70	0.998
**1859**	41	3.816	6.6	15 - 70	0.996
**1859**	41	4.762	8.2	7.5 - 80	0.992
**1859**	**41**	**n < 4**			
**1855**	32	2.885	52.0	20 - 100	1.000
**1855**	32	3.142	54.0	20 - 100	1.000
**1855**	32	2.885	55.6	20 - 100	0.999
**1855**	32	3.129	59.5	20 - 100	0.999
**1855**	32	3.446	94.5	20 - 100	0.999
**1855**	**32**	**3.010**			**1.000**
		**0.145**			

**1843**	22	3.286	22.1	10 - 100	0.997
**1843**	22	3.779	27.3	10 - 100	0.999
**1843**	22	3.849	27.5	10 - 100	1.000
**1843**	22	3.112	27.9	10 - 100	0.996
**1843**	22	3.823	28.7	10 - 100	0.995
**1843**	22	3.726	35.7	15 - 100	0.996
**1843**	22	3.569	37.7	15 - 100	0.999
**1843**	22	3.800	38.5	15 - 100	1.000
**1843**	22	3.735	38.7	15 - 100	0.999
**1843**	22	3.733	39.4	15 - 100	0.996
**1843**	**22**	**3.681**			**0.998**
		**0.228**			
**1800**	42	3.605	8.3	15 - 100	0.990
**1800**	42	3.481	8.3	15 - 100	0.987
**1800**	42	3.463	8.4	15 - 100	0.993
**1800**	42	3.530	8.7	15 - 100	0.991
**1800**	42	2.532	10.0	15 - 100	0.993
**1800**	40	2.670	11.0	10 - 100	0.989
**1800**	40	2.684	13.6	10 - 100	0.998
**1800**	40	2.616	14.1	10 - 100	0.992
**1800**	40	2.459	14.2	10 - 100	0.991
**1800**	43	2.682	14.3	15 - 100	0.994
**1800**	43	2.602	14.6	15 - 100	0.981
**1800**	43	2.662	14.8	15 - 100	0.980
**1800**	43	2.741	14.9	15 - 100	0.990
**1800**	43	2.466	24.5	15 - 100	0.951
**1800**	**multiple**	**n < 4**			
**1790**	20	5.320	16.3	15 - 100	0.997
**1790**	20	5.683	22.6	15 - 100	0.997
**1790**	20	3.843	23.0	15 - 100	0.997
**1790**	20	4.224	23.8	15 - 100	0.997
**1790**	20	3.220	24.5	15 - 100	0.999
**1790**	20	4.209	31.8	15 - 100	0.998
**1790**	20	4.832	34.3	15 - 100	0.993
**1790**	20	4.563	35.4	15 - 100	0.997
**1790**	**20**	**4.149**			**0.997**
		**0.566**			
**1554**	8	3.699	43.4	10 - 100	0.999
**1554**	8	5.439	73.9	10 - 100	0.997
**1554**	8	5.563	75.8	10 - 100	0.983
**1554**	8	4.806	77.9	10 - 100	0.995
**1554**	8	4.823	82.2	10 - 100	0.966
**1554**	8	5.443	88.9	10 - 100	0.987
**1554**	**8**	**n < 4**			
**1530**	35	4.451	33.4	25 - 100	0.998
**1530**	35	3.637	34.7	25 - 100	0.999
**1530**	35	2.990	42.2	25 - 100	0.997
**1530**	35	3.317	43.8	25 - 100	0.998
**1530**	35	2.996	46.2	25 - 100	0.998
**1530**	35	2.624	52.1	25 - 100	0.996
**1530**	**35**	**3.336**			**0.998**
		**0.645**			
**1424**	21	3.669	23.7	10 - 100	0.997
**1424**	21	3.780	43.3	10 - 100	0.999

**1424**	21	3.928	49.8	10 - 100	0.988
**1424**	21	4.141	53.7	10 - 100	0.991
**1424**	21	4.186	53.7	10 - 100	0.998
**1424**	21	4.263	70.5	10 - 100	0.987
**1424**	**21**	**3.941**			**0.995**
		**0.224**			
**1380**	9	3.536	29.7	10 - 100	0.997
**1380**	9	3.898	38.2	10 - 100	1.000
**1380**	9	4.068	38.5	10 - 100	0.999
**1380**	9	3.577	40.7	10 - 100	0.998
**1380**	9	3.475	46.7	10 - 100	0.996
**1380**	9	3.581	47.7	10 - 100	0.994
**1380**	**9**	**3.689**			**0.997**
		**0.237**			
**1379**	29	3.782	15.9	10 - 100	0.999
**1379**	29	4.240	16.7	10 - 100	1.000
**1379**	29	3.798	17.9	10 - 100	0.999
**1379**	29	3.246	20.0	10 - 100	0.997
**1379**	29	3.161	21.3	10 - 100	0.998
**1379**	29	3.911	21.8	10 - 100	0.998
**1379**	**29**	**n < 4**			
**1323**	25	4.889	27.2	20 - 100	0.995
**1323**	25	4.792	27.6	20 - 100	0.999
**1323**	25	4.573	31.7	20 - 100	0.996
**1323**	25	4.382	32.2	20 - 100	0.999
**1323**	**25**	**4.659**			**0.997**
		**0.227**			
**1264**	34	2.814	13.4	10 - 100	0.999
**1264**	34	2.850	13.7	10 - 100	0.998
**1264**	34	2.847	14.0	10 - 100	1.000
**1264**	34	3.315	14.1	10 - 100	0.998
**1264**	34	3.072	16.6	10 - 100	0.998
**1264**	34	3.339	19.0	10 - 100	0.999
**1264**	**34**	**n < 4**			
**1135**	27A	5.621	19.3	10 - 100	0.996
**1135**	27A	5.681	19.8	10 - 100	0.998
**1135**	27A	5.732	21.0	10 - 100	0.998
**1135**	27A	5.881	21.3	10 - 100	0.996
**1135**	27A	5.897	22.5	10 - 100	0.998
**1135**	27A	5.933	24.3	15- 70	0.999
**1135**	27B	4.907	25.2	15- 70	0.999
**1135**	27B	4.879	25.7	15- 70	0.994
**1135**	27B	5.170	28.6	15- 70	0.999
**1135**	27B	5.001	28.9	15- 70	1.000
**1135**	27B	5.197	29.5	15- 70	0.999
**1135**	**27**	**5.181**			**0.998**
		**0.391**			
**1090**	26	6.344	23.0	20 - 150	0.997
**1090**	26	6.348	26.2	20 - 150	0.998
**1090**	26	6.847	26.7	20 - 150	0.994
**1090**	26	6.562	26.7	20 - 150	0.994
**1090**	26	6.850	26.8	20 - 150	0.995
**1090**	26	6.836	28.2	20 - 150	0.995

**1090**	**26**	**6.631**			**0.995**
		**0.247**			
**924**	28	2.836	7.2	10 - 100	0.999
**924**	28	2.826	7.4	10 - 100	0.998
**924**	28	2.800	7.7	10 - 100	1.000
**924**	28	2.629	8.3	10 - 100	0.999
**924**	28	2.939	8.6	10 - 100	0.999
**924**	28	2.237	9.0	10 - 100	1.000
**924**	**28**	**n < 4**			
**747**	11	2.389	7.6	10 - 100	0.999
**747**	11	2.470	7.9	10 - 100	0.998
**747**	11	2.523	8.2	10 - 100	1.000
**747**	11	2.444	8.2	10 - 100	0.999
**747**	11	2.391	8.4	10 - 100	0.999
**747**	11	2.595	8.5	10 - 100	0.997
**747**	**11**	**n < 4**			
**670**	16	1.800	13.4	10 - 100	0.997
**670**	16	1.881	13.4	10 - 100	0.993
**670**	16	1.842	13.4	10 - 100	0.992
**670**	16	1.933	13.5	10 - 100	0.998
**670**	16	1.948	13.6	10 - 100	0.993
**670**	16	1.884	13.7	10 - 100	0.999
**670**	**16**	**n < 4**			
**588**	30G	1.220	9.9	15 - 100	0.994
**588**	30F	1.634	10.2	5 - 50	0.990
**588**	30F	1.632	10.6	5 - 50	0.993
**588**	30G	1.051	10.9	15 - 100	0.985
**588**	30G	0.977	10.9	15 - 100	0.938
**588**	30G	0.819	11.1	15 - 100	0.957
**588**	30F	1.697	11.2	5 - 50	0.981
**588**	30F	1.728	11.3	5 - 50	0.971
**588**	30F	1.423	11.5	5 - 50	0.962
**588**	30F	1.423	12.6	5 - 50	0.994
**588**	30G	2.219	12.8	15 - 100	0.997
**588**	30D	1.860	13.0	5 - 100	0.993
**588**	30G	0.729	13.0	15 - 100	0.983
**588**	10	3.255	20.5	10 - 100	0.999
**588**	10	3.316	21.2	10 - 100	0.995
**588**	10	3.35	21.7	10 - 100	0.996
**588**	10	3.326	21.8	10 - 100	0.997
**588**	10	3.322	22.8	10 - 100	0.992
**588**	10	3.395	23.4	10 - 100	0.99
**588**	30D	3.320	25.5	5 - 50	0.991
**588**	30C	4.070	29.5	10 - 100	0.983
**588**	30E	4.070	29.5	10 - 80	0.992
**588**	30C	3.987	30.0	10 - 100	0.989
**588**	30E	3.987	30.0	10 - 80	0.989
**588**	30C	4.713	30.4	10 - 100	0.999
**588**	30E	4.713	30.4	10 - 80	0.977
**588**	30B	3.808	32.3	10 - 100	0.999
**588**	30B	3.765	33.2	10 - 100	0.991
**588**	30C	4.269	34.7	10 - 100	0.998
**588**	30E	4.269	34.7	10 - 80	0.995
**588**	30B	3.764	34.7	10 - 100	0.999
**588**	30B	4.226	35.3	10 - 100	0.998

**588**	30B	4.180	35.4	10 - 100	0.998
**588**	30C	3.932	35.4	10 - 100	0.995
**588**	30E	3.932	35.4	10 - 80	0.991
**588**	30C	3.842	35.8	10 - 100	0.990
**588**	30E	3.842	35.8	10 - 80	0.992
**588**	30B	4.087	35.8	10 - 100	0.999
**588**	30D	3.963	44.1	5 - 50	0.989
**588**	30D	2.783	70.9	5 - 70	0.992
**588**	30D	3.074	70.9	5 - 70	0.997
**588**	30D	2.326	-	-	-
**588**	**multiple**	**4.006**			**0.993**
		**0.339**			
**463**	33	2.933	20.7	2.5 - 100	0.999
**463**	33	2.622	30.4	2.5 - 100	1.000
**463**	33	2.401	30.7	2.5 - 100	1.000
**463**	36	4.578	45.3	20 - 225	0.999
**463**	36	4.524	45.7	25 - 100	0.998
**463**	36	4.083	68.2	20 - 225	0.999
**463**	36	5.199	83.1	20 - 225	0.999
**463**	36	3.770	87.1	20 - 225	0.998
**463**	36	5.336	94.8	25 - 100	0.990
**463**	36	4.208	97.1	25 - 100	0.988
**463**	36	4.575	99.9	20 - 225	0.974
**463**	36	3.450	108.7	25 - 100	0.981
**463**	**multiple**	**3.532**			**0.999**
		**1.181**			
**425**	45	3.182	47.2	10 - 100	0.998
**425**	45	2.945	50.8	10 - 100	0.999
**425**	45	2.982	51.8	10 - 100	0.997
**425**	45	3.123	53.5	10 - 100	0.998
**425**	45	3.118	54.5	10 - 100	0.999
**425**	45	3.402	84.8	10 - 100	0.999
**425**	**45**	**3.070**			**0.998**
		**0.101**			

Supplementary Table S7 – Summary of pseudo-Thellier results.

Per age group (first column), the following parameters are specified: the reference intensity (if applicable, GUFM1 between 1840 and 1900 AD, IGRF after 1900 AD); the average pseudo-Thellier slope with its standard deviation; and the calibrated absolute archaeointensity, together with its standard deviation. Out of the 33 age groups sampled in this study, 20 cooling units yielded a reliable calibrated archaeointensity estimate.

date AD	reference intensity	pseudo-Thellier slope	standard deviation	calibrated intensity [μT]	standard deviation [μT]
**2009**	34.7	n < 4			
**1990**	35.3	2.567	0.448	33.6	3.3
**1960**	36.1	3.033	0.237	37.0	1.7
**1955**	36.2	3.056	0.516	37.2	3.8
**1950**	36.2	3.023	0.122	36.9	0.9
**1942**	36.4	n < 4			
**1935**	36.5	2.700	0.222	34.6	1.6
**1926**	36.8	2.901	0.058	36.0	0.4
**1919**	37.1	2.826	0.054	35.4	0.4
**1907**	37.7	n < 4			
**1899**	38.2	n < 4			
**1887**	38.6	n < 4			
**1868**	39.1	3.327	0.373	39.2	2.7
**1859**	39.5	n < 4			
**1855**	39.6	3.010	0.145	36.9	1.1
**1843**	39.7	3.681	0.228	41.8	1.7
**1800**		n < 4			
**1790**		4.149	0.566	45.2	4.2
**1554**		n < 4			
**1530**		3.336	0.645	39.2	4.8
**1424**		3.941	0.224	43.7	1.6
**1380**		3.689	0.237	41.9	1.7
**1379**		n < 4			
**1323**		4.659	0.227	49.0	1.7
**1264**		n < 4			
**1135**		5.181	0.391	52.9	2.9
**1090**		6.631	0.247	63.5	1.8
**924**		n < 4			
**747**		n < 4			
**670**		n < 4			
**588**		4.006	0.339	44.2	2.5
**463**		3.532	1.181	40.7	8.7
**425**		3.070	0.101	37.3	0.7

Supplementary Table S8 – Accepted archaeointensity results from the GEOMAGIA database.
From left to right: the dating sample (if specified in the reporting study); laboratory ^{14}C ages (years BP, with error); median probability age (AD) obtained after recalibrating the ^{14}C-lab age with INTCAL.09; probability intervals of the recalibration (lower limit – upper limit; probability); number of independent intensity samples included in the average reported archaeointensity; archaeointensity; error in archaeointensity; and a reference to the reporting study.

Dating sample	^{14}C lab age	Median prob. age	Calibration intervals	n	Int.	St.dev.	Ref.
**W4344**	1690 ± 210	330	126 – 570; 1	4	39.3	3.1	10
**W3857**<br>**W3858**	1470 ± 60<br>1400 ± 60	608	547 – 673; 1	5	36.8	6.8	9
**W4237**	1320 ± 150	727	593 – 888; 1	4	42.4	3.6	10
**W5522**	1180 ± 120	846	711 – 746; 0.128<br>766 – 974; 0.872	5	41.4	3.89	12
**?**	1058 ± 50	977	899 – 919; 0.187<br>952 – 1022; 0.813	4	63.9	6.43	12
**W4047**	910 ± 70	1121	1037 – 1180; 1	5	61.48	2.97	12
**?**	900 ± 120	1122	1023 – 1225; 1	5	63.87	4.15	12
**W3879**	830 ± 60	1197	1159 – 1265; 1	5	49.6	4.6	9
**W3881**	260 ± 210	1655	1454 – 1694; 0.654<br>1727 – 1813; 0.229<br>1839 – 1841; 0.117<br>1853 – 1859; 0.012<br>1861 – 1867; 0.012<br>1918 – 1952; 0.086		39.3	3.1	10
	historical	1840		5	35.01	2.0	9
	historical	1955		4	42.4	6.9	11
	historical	1960		5	31.8	3.4	27
	historical	1960		5	38	6	27
	historical	1977		4	37.8	1.9	11
	historical	1982		6	38.2	2.1	11

Supplementary Table S9 – Accepted archaeodirections from the GEOMAGIA database.
All directions are from Hagstrum & Champion[35]. From left to right: the dating sample, the ^{14}C laboratory age, the recalibrated (INTCAL.09) age AD, and the declination and inclination.

Dating sample	^{14}C lab age	Calibrated age AD	Declination	Inclination
**W-6264**	1920 ± 80	85	5.8	20.4
**W-5949**	1880 ± 200	125	-7.1	31.7
**W-4116**	1840 ± 60	175	-4.8	16.8
**W-3850**	1810 ± 80	210	-6.5	31.9
**W-3850**	1810 ± 80	210	-7.7	31.0
**W-5564**	1740 ± 250	270	-4.5	24.2
**W-5271**	1700 ± 80	338	8.0	20.2
**W-4344**	1690 ± 70	349	-4.4	18.0
**W-5458**	1640 ± 150	394	2.2	26.0
**W-5633**	1600 ± 200	425	6.6	25.5
**W-5788**	1590 ± 100	461	2.2	22.7
**W-5670**	1500 ± 200	527	3.0	16.6
**W-5989**	1500 ± 130	535	4.1	20.7
**W-4357**	1490 ± 50	572	-1.0	17.2
**W-4981**	1470 ± 50	588	-0.2	22.8
**W-3858**	1400 ± 60	633	4.3	24.3
**W-4340**	1400 ± 70	633	1.9	18.9
**W-5325**	1370 ± 200	670	8.9	20.9
**W-4383**	1330 ± 50	693	6.2	40.6
**W-4414**	1330 ± 60	699	8.4	21.1
**W-4237**	1320 ± 50	703	2.9	18.5
**W-4237**	1320 ± 50	703	2.2	18.2
**W-3000**	1330 ± 70	703	4.3	25.3
**W-4343**	1280 ± 70	747	0.1	18.9
**W-3910**	1270 ± 60	749	6.6	20.7
**W-4430**	1270 ± 70	754	6.4	21.3
**W-4674**	1270 ± 70	754	6.1	25.3
**W-5522**	1180 ± 200	853	-3.7	27.0
**W-6049**	1170 ± 100	853	3.2	31.2
**W-5791**	1170 ± 150	859	1.3	39.0
**W-5135**	1150 ± 70	877	-1.9	38.2
**W-5519**	1150 ± 200	880	-4.3	26.1
**W-5212**	1140 ± 70	889	-5.8	22.5
**W-3827**	1130 ± 60	905	-6.0	21.2
**W-5537**	1120 ± 200	907	-7.1	23.3
**W-5537**	1120 ± 200	907	-7.5	24.1
**W-4631**	1110 ± 60	924	0.6	21.4
**W-5625**	1100 ± 110	922	-5.0	29.1
**W-5319**	970 ± 120	1068	4.2	42.6
**W-5211**	960 ± 70	1090	6.1	21.1
**W-5975**	900 ± 120	1122	-1.2	37.5
**W-4047**	910 ± 70	1121	-0.6	36.9
**W-4047**	910 ± 70	1121	-3.7	37.9
**W-4690**	900 ± 70	1128	-2.7	40.5
**W-4137**	890 ± 60	1135	-0.5	40.4
**w-3879**	740 ± 60	1264	4.5	38.7
**W-5627**	750 ± 200	1230	-4.3	27.7
**W-4232**	780 ± 70	1232	-9.7	29.7
**W-4345**	760 ± 70	1247	-6.0	26.0
**w-5973**	740 ± 100	1258	4.0	41.1
**w-3999**	730 ± 80	1271	2.9	37.6
**w-4012**	740 ± 60	1264	-2.1	41.2
**w-4156**	740 ± 60	1264	0.2	38.0
**w-4402**	700 ± 70	1296	4.2	41.6
**w-3860**	670 ± 60	1323	1.8	37.5
**w-3860**	670 ± 60	1323	1.7	35.1

**w-4025**	640 ± 50	1345	-3.1	37.8
**w-4337**	620 ± 70	1347	0.7	41.9
**w-4338**	590 ± 50	1351	0.0	39.3
**w-5785**	600 ± 100	1353	4.0	38.7
**w-4118**	580 ± 80	1358	6.2	40.7
**w-5098**	570 ± 60	1357	5.1	40.2
**w-4404**	530 ± 70	1394	-0.6	38.5
**w-4404**	530 ± 70	1394	0.5	39.2
**w-5983**	550 ± 100	1382	6.9	40.3
**w-5983**	550 ± 100	1382	4.5	40.5
**w-5897**	550 ± 150	1397	4.4	35.6
**w-4688**	490 ± 60	1424	6.1	40.6
**w-5145**	500 ± 100	1424	4.9	40.1
**w-4234**	490 ± 80	1427	0.9	33.4
**w-5568**	510 ± 150	1431	2.7	37.5
**w-3793**	450 ± 60	1456	4.2	45.6
**w-3842**	450 ± 60	1456	7.6	36.5
**w-3842**	450 ± 60	1456	4.9	37.5
**w-3941**	450 ± 60	1456	3.3	41.2
**w-3941**	450 ± 60	1456	1.7	39.3
**w-5932**	462 ± 111	1465	0.2	39.4
**w-5932**	462 ± 111	1465	1.0	37.6
**w-4049**	420 ± 70	1495	-2.2	39.1
**w-5079**	410 ± 60	1499	4.1	38.1
**w-5803**	390 ± 100	1531	1.3	40.2
**w-5757**	410 ± 250	1542	4.2	37.2
**w-3870**	350 ± 60	1549	1.3	39.1
**w-3870**	350 ± 60	1549	3.5	35.6
**w-3870**	350 ± 60	1549	7.2	40.0
**w-3811**	330 ± 60	1558	3.0	37.9
**w-3811**	330 ± 60	1558	4.5	36.8
**w-3811**	330 ± 60	1558	3.3	38.5
**w-4238**	330 ± 70	1560	6.8	33.2
**w-4162**	310 ± 70	1571	2.7	43.1
**w-4162**	310 ± 70	1571	1.5	44.0
**w-4394**	300 ± 60	1572	-8.1	31.5
**w-5106**	300 ± 80	1584	5.4	41.2
**w-4006**	290 ± 70	1587	2.4	41.9
**w-3881**	260 ± 70	1640	2.0	43.3
**w-3881**	260 ± 70	1640	3.1	46.0
**w-3881**	260 ± 70	1640	3.2	44.2
**w-3881**	260 ± 70	1640	5.5	41.3
**w-3871**	230 ± 60	1726	-0.1	37.2
**w-3871**	230 ± 60	1726	1.4	37.8
	historical	1758	4.6	38.2
	historical	1750	8.1	37.2
	historical	1750	5.4	39.7
	historical	1750	6.4	34.8
	historical	1750	6.3	36.9
	historical	1750	6.3	33.6
	historical	1790	7.8	40.1
	historical	1790	6.7	39.4
	historical	1790	6.8	37.1
	historical	1790	8.7	37.3
	historical	1790	7.9	37.1
	historical	1790	7.7	38.5
	historical	1790	6.3	36.8
	historical	1790	8.1	37.3
	historical	1790	6.9	39.3
	historical	1790	8.9	39.1
	historical	1790	10.9	38.8
	historical	1790	6.8	36.8

historical	1800	12.0	34.8
historical	1840	6.1	35.7
historical	1840	5.2	40.4
historical	1840	5.9	40.0
historical	1840	6.8	37.5
historical	1843	10.6	35.1
historical	1852	8.3	35.1
historical	1859	10.7	36.4
historical	1868	9.1	33.8
historical	1868	6.8	33.2
historical	1881	7.5	33.9
historical	1907	10.6	36.2
historical	1919	15.0	30.0
historical	1919	5.6	29.1
historical	1921	12.6	28.4
historical	1926	9.9	33.8
historical	1935	15.3	37.5
historical	1940	15.1	37.3
historical	1950	9.1	33.8
historical	1955	10.7	34.8
historical	1955	13.3	34.4
historical	1955	11.8	33.8
historical	1955	13.6	38.4
historical	1960	14.7	34.5
historical	1960	10.9	35.2
